# Type 2 diabetes risks and determinants in second-generation migrants and mixed ethnicity people of South Asian and African Caribbean descent in the UK

**DOI:** 10.1007/s00125-021-05580-7

**Published:** 2021-10-20

**Authors:** Aliki-Eleni Farmaki, Victoria Garfield, Sophie V. Eastwood, Ruth E. Farmer, Rohini Mathur, Olga Giannakopoulou, Praveetha Patalay, Karoline Kuchenbaecker, Naveed Sattar, Alun Hughes, Krishnan Bhaskaran, Liam Smeeth, Nish Chaturvedi

**Affiliations:** 1 MRC Unit for Lifelong Health and Ageing, Institute of Cardiovascular Science, University College London, London, UK; 2 London School of Hygiene & Tropical Medicine, London, UK; 3 Division of Psychiatry, University College London, London, UK; 4 UCL Genetics Institute, University College London, London, UK; 5 Centre for Longitudinal Studies, University College London, London, UK; 6 Institute of Cardiovascular and Medical Sciences, University of Glasgow, Glasgow, UK

**Keywords:** African Caribbeans, Genetic admixture, Migrants, Mixed populations, Second generation, South Asians, Type 2 diabetes, UK Biobank

## Abstract

**Aims/hypothesis:**

Excess risks of type 2 diabetes in UK South Asians (SA) and African Caribbeans (AC) compared with Europeans remain unexplained. We studied risks and determinants of type 2 diabetes in first- and second-generation (born in the UK) migrants, and in those of mixed ethnicity.

**Methods:**

Data from the UK Biobank, a population-based cohort of ~500,000 participants aged 40–69 at recruitment, were used. Type 2 diabetes was assigned using self-report and HbA_1c_. Ethnicity was both self-reported and genetically assigned using admixture level scores. European, mixed European/South Asian (MixESA), mixed European/African Caribbean (MixEAC), SA and AC groups were analysed, matched for age and sex to enable comparison. In the frames of this cross-sectional study, we compared type 2 diabetes in second- vs first-generation migrants, and mixed ethnicity vs non-mixed groups. Risks and explanations were analysed using logistic regression and mediation analysis, respectively.

**Results:**

Type 2 diabetes prevalence was markedly elevated in SA (599/3317 = 18%) and AC (534/4180 = 13%) compared with Europeans (140/3324 = 4%). Prevalence was lower in second- vs first-generation SA (124/1115 = 11% vs 155/1115 = 14%) and AC (163/2200 = 7% vs 227/2200 = 10%). Favourable adiposity (i.e. lower waist/hip ratio or BMI) contributed to lower risk in second-generation migrants. Type 2 diabetes in mixed populations (MixESA: 52/831 = 6%, MixEAC: 70/1045 = 7%) was lower than in comparator ethnic groups (SA: 18%, AC: 13%) and higher than in Europeans (4%). Greater socioeconomic deprivation accounted for 17% and 42% of the excess type 2 diabetes risk in MixESA and MixEAC compared with Europeans, respectively. Replacing self-reported with genetically assigned ethnicity corroborated the mixed ethnicity analysis.

**Conclusions/interpretation:**

Type 2 diabetes risks in second-generation SA and AC migrants are a fifth lower than in first-generation migrants. Mixed ethnicity risks were markedly lower than SA and AC groups, though remaining higher than in Europeans. Distribution of environmental risk factors, largely obesity and socioeconomic status, appears to play a key role in accounting for ethnic differences in type 2 diabetes risk.

**Graphical abstract:**

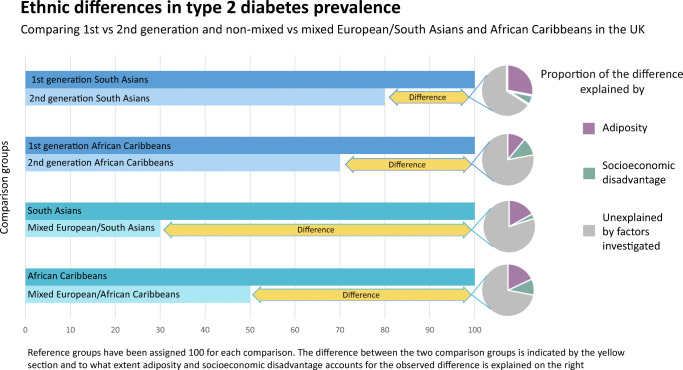

**Supplementary Information:**

The online version contains peer-reviewed but unedited supplementary material available at 10.1007/s00125-021-05580-7.

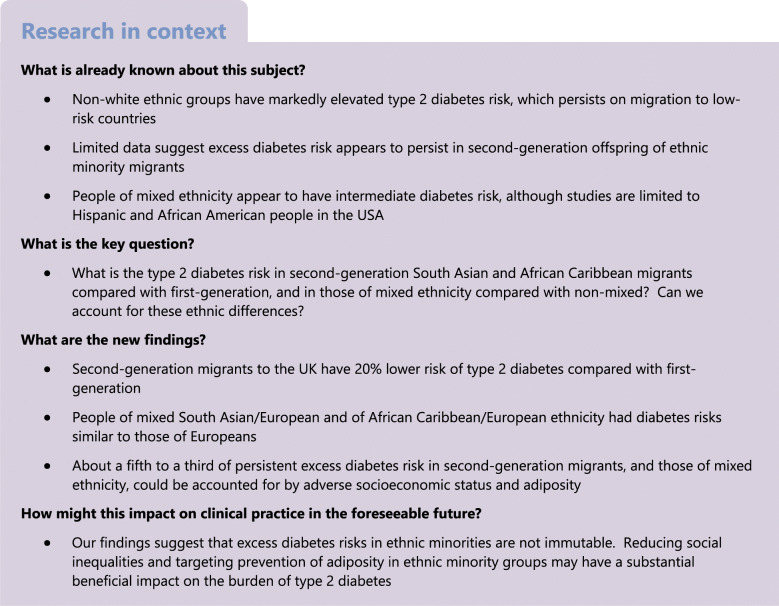



## Introduction

Type 2 diabetes is estimated to affect 693 million people worldwide by 2045 [1]. People of African Caribbean (AC) and South Asian (SA) descent have some of the highest rates of type 2 diabetes in the world, often three to four times greater, respectively, than those of European ancestry when compared in the same setting [2]. Explanations for this excess risk, both environmental and genetic, remain unclear. We have previously reported that adiposity can only account for part of the excess diabetes risk [2]. Others have observed population-specific variants in genes implicated in insulin signalling, adipogenesis and energy conservation in SA [3], and for beta cell mass and insulin response in people of African descent [4]. However, these differences are insufficient to account for the excess diabetes risk [5, 6].

Studies of migrant offspring, and people of mixed ethnicity, where distribution and inter-relations of genetic and environmental explanatory factors differ, may offer fresh insights. Previous studies suggest second and subsequent generations of migrants are at persistently elevated risk [7–10]. Partial European ancestry has been associated with decreased and non-European ancestry with increased type 2 diabetes risk in admixed populations of Hispanic, African American, East Asian and European descent [11–15]. Using genetic admixture approaches to define ancestry, some or all of the excess diabetes risk in people of African American or Hispanic American descent compared with European descent was explained by socioeconomic status (SES) and adiposity [16, 17]. The majority of these genetic admixture studies have been performed in the USA, where the correlation between race/ethnicity and SES is high, making it difficult to dissect individual contributions.

To date, no study has combined mixed ethnicity comparisons and inter-generational analysis in the same setting to understand the impact of mutable environmental risk factors in determining risk. First- and second-generation migrants have similar genetic makeup and differ mainly in terms of environmental exposures, while mixed ethnic groups are likely to have different genetic backgrounds and environmental exposures to non-mixed groups.

Our primary hypothesis was that people of mixed European/South Asian (MixESA) or mixed European/African Caribbean (MixEAC) ethnicity would have risks of type 2 diabetes intermediate between each of the parental ethnic groups, while offspring whose parents were both migrants of either SA or AC ethnicity would retain the excess parental risks of type 2 diabetes. The secondary hypothesis was that observed ethnic differences in type 2 diabetes would largely be accounted for by differences in adiposity, lifestyle and SES. We tested these hypotheses in the UK Biobank (UKB) cohort, which included relatively large numbers of people of both mixed ethnicities and second-generation offspring, born in the UK, of first-generation migrants.

## Methods

### Study design

UKB is a large population-based cohort of over 500,000 men and women aged 40–69 years recruited from primary care lists in the UK between 2006 and 2010 [18]. The following data were collected by self-completion or nurse-administered questionnaires at the recruitment clinic visit: self-defined ethnicity using the UK census classification [19]; year of migration to the UK to assign generational status; health behaviours including smoking (ever smoked), physical activity (number of days/week of moderate physical activity more than 10 min) and diet (data from the touchscreen questionnaire on the reported frequency of intake of a range of common food and drink items); and sociodemographic variables such as education and Townsend deprivation score assigned by residential postcode [20]. Height, weight and body circumferences were measured directly, and bio-impedance was used to assess fat mass and fat percentage (%). Participants were asked to recall birthweight.

A blood sample was taken for DNA extraction and measurement of biochemical markers in serum. HbA_1c_ (mmol/mol) was measured from a blood sample taken at the time of the visit, regardless of type 2 diabetes status. Values above 195 mmol/mol (20%) (*n* = 5) were considered outliers and excluded from the analysis.

‘Known type 2 diabetes’ at recruitment was defined according to an algorithm based on self-report data and medication; this algorithm has been validated against primary care records [21]. ‘All type 2 diabetes’ included those with ‘known type 2 diabetes’ plus all those with an HbA_1c_ > 47 mmol/mol (6.4%).

Migration status (first or second generation) was defined based on response to the question: ‘What year did you first come to live in the UK?’ This was completed by participants who indicated they were born outside the UK. Those from the ethnic minority group of interest reporting that they had been born outside the UK and reporting a year of migration were classified as first-generation migrants; otherwise, they were classified as second generation. Self-reported MixESA and MixEAC were the ethnic groups of interest, with European, SA and AC ethnicities for comparison.

We conducted principal component analysis (PCA) to identify underlying dietary patterns (electronic supplementary material [ESM] Methods, Dietary patterns). Food frequency dietary data from the touchscreen questionnaire at the recruitment assessment were used and the scores of the emerging dietary patterns were used in multivariable models.

### Matching procedure

We matched by sex and age, as ethnic minority populations in UKB are younger than the general European origin population; in addition, we wished to compare first- (born abroad, migrated to the UK) and second-generation (born to two ethnic minority parents, resident in the UK) migrants. The reference group was the mixed or the second-generation group, depending on the comparison. As the reference groups were the smallest, we matched to optimise power, employing 1:4 matching where possible, and 1:2 where the sample was insufficient (for the second- with first-generation migrant comparison). Matching was performed at random within sex and 5 year age bands. Each matching procedure was performed independently to create unique datasets for each analysis:

- MixESA (*n* = 831)–SA–Europeans (1:4:4), *N* = 7472
- MixEAC (*n* = 1045)–AC–Europeans (1:4:4), *N* = 9405
- Second-generation SA (*n* = 1115)–first-generation SA–Europeans (1:1:2), *N* = 4460
- Second-generation AC (*n* = 2200)–first-generation AC–Europeans (1:1:2), *N* = 8800

Details of the matching procedure and frequency distributions for each of the derived datasets are shown in ESM Figs 1–4. There was low overlap (<5%) between the European control participants selected for the different comparison groups, leading to minimal dependence between analyses (ESM Fig. 5).

### Statistical analyses

We explored the contribution of major risk factors in accounting for ethnic differences in type 2 diabetes. These included: smoking; Townsend deprivation score as a proxy for SES; height (cm); birthweight (kg); years of education derived from qualifications based on the International Standard Classification of Education (ISCED) coding [22]; and adiposity measures. We selected waist/hip ratio (WHR) as our key measure for adiposity in the SA analyses, and BMI for the AC analyses, as these measures best accounted for ethnic differences in type 2 diabetes in a previous population cohort analysis [2].

Multivariable logistic regression was used to examine the contribution of each different risk factor in the association of ethnicity with type 2 diabetes. Specifically, six different models were applied: Model 1: adjusted for age and sex; Model 2: Model 1 plus WHR (for SA) or BMI (for AC); Model 3: Model 1 plus deprivation index; Model 4: Model 1 plus ever smoking; Model 5: Model 1 plus height; Model 6: Model 1 plus years of education.

The extent to which adiposity patterns, deprivation, smoking, height and education mediated the relationship between ethnicity and type 2 diabetes was explored in path models. The path analysis is a form of multiple regression statistical analysis that is used to evaluate causal models by examining the relationships between a dependent variable and two or more independent variables. Path analysis allows the simultaneous modelling of several related regression relationships. By using this method, one can estimate both the magnitude and significance of causal connections between variables. In path analysis, a variable can be a dependent variable in one relationship and an independent variable in another. These variables are referred to as mediating variables. While path analysis is useful for evaluating causal hypotheses, this method cannot determine the direction of causality.

**Fig. 1:**
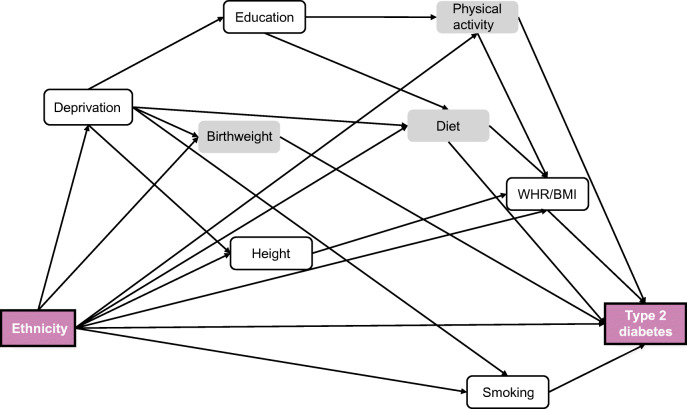
DAG of ethnicity on type 2 diabetes, including all the potential determinants of this relationship in age-/sex-matched individuals. The grey-coloured variables have not been carried forward to the subsequent analyses

A path model is a graph that shows the independent, intermediate and dependent variables [23, 24]. A path model can have three types of effects: the total effect, i.e. the observed effect of ethnicity on type 2 diabetes without adjustment for an intermediate variable; an indirect effect attributable to the intermediate variable; and the direct effect, i.e. the (independent) effect remaining of ethnicity on type 2 diabetes after adjustment for all intermediate variables depicted in the directed acyclic graph (DAG) (Fig. 1). The indirect effect is attributed to each of the mediators singly or jointly following the DAG-defined pathways and depends on the comparison made in the exposure, i.e. ethnicity. The indirect effect can therefore be expressed as the percentage of the total effect mediated by these explanatory variables. All models were adjusted for the potential confounders, age and sex [25].

Statistical analyses comparing recruitment characteristics were performed in Stata 15 [26]. Mediation analysis testing path models was performed with Mplus version 8.3 using the maximum likelihood ratio estimator and Monte Carlo integration at 10,000 [27].

Sample sizes by comparison group for different analyses are shown in ESM Fig. 6.

### Admixture definition

In total, 38,598 non-European participants remained in the dataset after quality control (ESM Methods, Genetic data quality control). We estimated principal components (PCs) for these participants using PC-Air implemented in the GENESIS package; this has been optimised for samples with population admixture [28]. We initially used clustering with five *k*-means (to correspond to the five main self-reported ethnicities in UKB) on the non-European sample in order to identify and remove individuals with East Asian ancestry (self-reported as Chinese) (ESM Fig. 7). Ten *k*-means best identified the discreet Asian/African admixed group (cluster no. 3), which was then excluded from subsequent analyses (ESM Fig. 8). We retained nine clusters: five for the SA admixture analysis and five for the AC admixture analysis, with one common cluster (cluster no. 10). We identified centroids for each of the SA, AC and White British (European) clusters, applying *k*-means to the GENESIS PCs for SA and AC, and to UKB PCs for Europeans (ESM Fig. 9). A genetic admixture score from 0% (European) to 100% (SA/AC) was assigned to all participants included in our mixed ethnicity analysis (MixESA–SA–Europeans, *N* = 7472; and MixEAC–AC–Europeans, *N* = 9405) based on the distance of that individual from the European centroid as a proportion of the total distance between the European–SA/AC centroids.

The distribution of admixture level across the different groups of comparison i.e. MixESA–SA–Europeans and MixEAC–AC–Europeans have been tested with Kolmogorov–Smirnov and found to be significantly different (*p* < 0.001).

Level of admixture (%) was treated as a continuous variable and modelled using fractional polynomials with percentage admixture as the explanatory variable and type 2 diabetes as outcome, adjusting for all covariates used in the mediation models: age, sex, smoking, deprivation, height, years of education and adiposity. Fractional polynomials of power (1) provided the best fit to the model.

### Sensitivity analyses

Our initial DAG included physical activity and diet (Fig. 1, ESM Fig. 10). We performed sensitivity analyses using standard regression techniques to determine the impact of physical activity and diet in accounting for ethnic differences in diabetes risk and the importance of birthweight as a covariate. Sensitivity analyses were also performed using: (1) BMI in SA and WHR for AC; and (2) replacing type 2 diabetes with HbA_1c_ as the outcome. The multivariable analysis was also performed on ‘known’ type 2 diabetes without including individuals with type 2 diabetes based on HbA_1c_. Additionally, we have undertaken a sensitivity analysis with HbA_1c_ as an outcome after excluding all those with diagnosed (‘known’) type 2 diabetes.

### Missing data

Data missingness in all the matched sets was very low, as follows: smoking <0.5%; deprivation <0.5%; WHR <2%; BMI <3%; education <7%; and height <3%. For physical activity and diet, missingness was <17%, while for birthweight it was >50%. Physical activity and birthweight as predictors of diabetes prevalence were therefore only used in sensitivity analysis given the high level of missingness.

### Ethics approval

UKB has approval from the North West Multi-centre Research Ethics Committee (MREC), which covers the UK. In Scotland, UKB has approval from the Community Health Index Advisory Group (CHIAG).

## Results

### Ethnic differences in type 2 diabetes prevalence

**Fig. 2:**
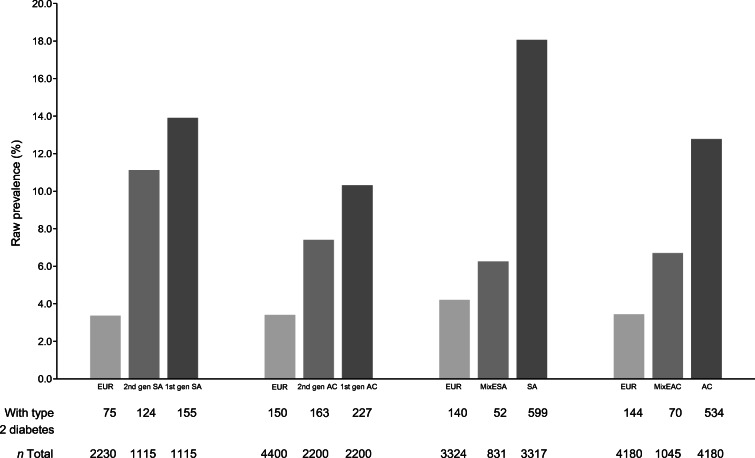
Bar chart for type 2 diabetes prevalence by ethnic group. EUR, Europeans; gen, generation

Overall, type 2 diabetes prevalence was almost fivefold higher in SA (18%), and threefold higher in AC (13%), than in Europeans (4%) (Fig. 2). Type 2 diabetes prevalence in those of mixed ethnicity was lower than in non-mixed. MixESA (6%) had one-third the diabetes prevalence of SA (18%), and MixEAC (7%) prevalence was half that of AC (13%). Second-generation SA (11%) and AC (7%) had ~20% and ~30%, respectively, lower prevalence of type 2 diabetes than first-generation migrants (SA 14%, AC 10%) (Fig. 2). However, both second-generation migrants and those of mixed ethnicity had persistently elevated type 2 diabetes prevalence compared with Europeans.

### Ethnic differences in key risk factors for diabetes

WHR was higher in SA (0.89) than in Europeans (0.86) (Tables 1 and 2). While WHR in MixESA was identical to that in Europeans (0.86), WHR in second-generation SA (0.88), though lower than in first-generation SA, remained elevated compared with Europeans. Socioeconomic deprivation followed a similar pattern. Residence in the most deprived quintile was nearly twice as high in SA (37%) than in Europeans (19%), and remained elevated in those of mixed ethnicity (31%) and in second-generation SA (38%). Years of education was also lower in SA than in Europeans, apart from in MixESA, in which it was on average 0.7 years longer than in Europeans. Ever-smoking rates were favourable in SA, except in those of mixed ethnicity, which, at 37%, were higher than in Europeans (33%).

**Table 1:** Characteristics of UKB participants by ethnicity: European and SA origin groups by generation

Characteristic	European			SA second generation			SA first generation		
	All	Men	Women	All	Men	Women	All	Men	Women
*n* (%)	2230	1072 (48)	1158 (52)	1115	536 (48)	579 (52)	1115	536 (48)	579 (52)
Age, years	47.1 ± 6.7	46.6 ± 6.4	47.5 ± 6.9	46.6 ± 6.7	46.2 ± 6.5	47.0 ± 6.9	46.9 ± 6.7	46.5 ± 6.5	47.3 ± 6.9
Ever smoked	667 (30)	362 (34)	305 (26)	197 (18)	140 (26)	57 (10)	155 (14)	133 (25)	22 (4)
Most deprived Townsend quintile	457 (20)	229 (21)	228 (20)	425 (38)	209 (39)	216 (37)	450 (40)	244 (46)	206 (36)
Years of education derived	15.8 ± 0.7	15.8 ± 0.3	15.8 ± 1.0	15.0 ± 1.3	15.1 ± 1.4	14.8 ± 1.2	15.3 ± 0.8	15.8 ± 0.5	14.8 ± 0.7
WHR	0.86 ± 0.09	0.92 ± 0.07	0.80 ± 0.07	0.88 ± 0.09	0.93 ± 0.06	0.83 ± 0.08	0.89 ± 0.09	0.94 ± 0.06	0.84 ± 0.08
Height, cm	170 ± 10	177 ± 7	164 ± 7	165 ± 9	172 ± 7	159 ± 6	164 ± 9	171 ± 6	158 ± 6
BMI, kg/m^2^	27 ± 5	28 ± 4	26 ± 5	27 ± 5	28 ± 4	27 ± 6	27 ± 5	27 ± 4	27 ± 5
Fat, %	29.7 ± 8.5	24.1 ± 5.9	35.0 ± 7.2	31.5 ± 8.4	25.5 ± 5.3	37.1 ± 6.8	31.7 ± 8.2	25.5 ± 5.0	37.5 ± 6.0
Birthweight, kg	3.3 ± 0.6	3.4 ± 0.6	3.3 ± 0.6	3.1 ± 0.6	3.2 ± 0.7	3.0 ± 0.6	3.0 ± 0.7	3.1 ± 0.7	3.0 ± 0.7
Known type 2 diabetes	48 (2)	30 (3)	18 (2)	91 (8)	53 (10)	38 (7)	115 (10)	65 (12)	50 (9)
All type 2 diabetes^a^	75 (3)	43 (4)	32 (3)	124 (11)	71 (13)	53 (9)	155 (14)	92 (17)	63 (11)
HbA_1c_, mmol/mol	34.5 ± 1.4	35.1 ± 0.7	34.0 ± 1.6	38.2 ± 2.5	39.0 ± 1.9	37.5 ± 2.8	39.1 ± 2.3	39.8 ± 1.9	38.4 ± 2.5
HbA_1c_, %	5.3 ± 2.3	5.4 ± 2.2	5.3 ± 2.3	5.7 ± 2.4	5.7 ± 2.3	5.6 ± 2.4	5.7 ± 2.4	5.8 ± 2.3	5.7 ± 2.4
Glucose, mmol/l	4.97 ± 0.16	5.06 ± 0.12	4.89 ± 0.15	5.18 ± 0.27	5.29 ± 0.20	5.08 ± 0.29	5.23 ± 0.32	5.26 ± 0.31	5.21 ± 0.33

Data are *n* (%) and mean ± SD

First/second generation assigned by year of migration. European and SA first- and second-generation groups are age and sex matched (2:1:1). Numbers of Europeans differ between Tables 1 and 2 as a consequence of different matching ratios

^a^Includes known (algorithmically defined) diabetes and HbA_1c_ > 47 mmol/mol (6.4%)

**Table 2:** Characteristics of UKB participants by ethnicity: European and SA origin groups by admixture

Characteristic	European			MixESA			SA		
	All	Men	Women	All	Men	Women	All	Men	Women
*n* (%)	3324	1392 (41.9)	1932 (58.1)	831	348 (41.9)	483 (58.1)	3317	1392 (42)	1925 (58)
Mean SA admixture, %	0.6 ± 3.6	0.7 ± 4.0	0.6 ± 3.4	48.0 ± 21.6	49.6 ± 22.0	46.8 ± 21.2	77.5 ± 12.3	77.4 ± 12.2	77.6 ± 12.3
Age, years	52.3 ± 8.5	51.7 ± 8.4	52.6 ± 8.5	52.2 ± 8.5	51.5 ± 8.4	52.6 ± 8.5	52.1 ± 8.5	51.6 ± 8.5	52.5 ± 8.5
Ever smoked	1092 (33)	516 (37)	576 (30)	309 (37)	141 (40.5)	168 (35)	472 (14)	367 (26.4)	105 (5.5)
Most deprived Townsend quintile	622 (19)	248 (18)	374 (19)	259 (31)	115 (33)	144 (30)	1239 (37)	570 (41)	669 (35)
Years of education derived	15.2 ± 1.0	15.3 ± 0. 9	15.2 ± 1.2	15.9 ± 1.4	16.0 ± 1.4	15.9 ± 1.4	14.9 ± 0.7	15.4 ± 0.4	14.6 ± 0.6
WHR	0.86 ± 0.09	0.93 ± 0.06	0.81 ± 0.07	0.86 ± 0.09	0.93 ± 0.07	0.82 ± 0.07	0.89 ± 0.09	0.95 ± 0.06	0.85 ± 0.07
Height, cm	169 ± 9	176 ± 7	163 ± 6	167 ± 9	174 ± 7	161 ± 6	163 ± 9	171 ± 7	157 ± 6
BMI, kg/m^2^	27.2 ± 4.9	27.6 ± 4.3	26.9 ± 5.3	26.8 ± 4.9	27.2 ± 4.4	26.5 ± 5.3	27.4 ± 4.7	27.1 ± 4.1	27.7 ± 5.0
Fat, %	31 ± 9	24 ± 6	36 ± 7	31 ± 9	24.5 ± 6	36 ± 7	33 ± 8	26 ± 5	38 ± 6
Birthweight, kg	3.3 ± 0.6	3.4 ± 0.7	3.2 ± 0.6	3.2 ± 0.7	3.2 ± 0.7	3.1 ± 0.6	3.0 ± 0.7	3.2 ± 0.7	3.0 ± 0.7
Known type 2 diabetes	107 (3)	74 (5)	33 (2)	39 (5)	25 (7)	14 (3)	477 (14)	243 (17)	234 (12)
All type 2 diabetes^a^	140 (4)	88 (6)	52 (3)	52 (6)	33 (9)	19 (4)	599 (18)	299 (21)	300 (16)
HbA_1c_, mmol/mol	35.1 ± 1.7	35.5 ± 1.5	34.8 ± 1.7	36.6 ± 2.2	37.8 ± 2.1	35.8 ± 2.0	40.2 ± 2.6	41.0 ± 2.5	39.6 ± 2.5
HbA_1c_, %	5.4 ± 2.3	5.4 ± 2.3	5.3 ± 2.3	5.5 ± 2.4	5.5 ± 2.3	5.4 ± 2.3	5.8 ± 2.4	5.9 ± 2.4	5.8 ± 2.4
Glucose, mmol/l	5.01 ± 0.14	5.07 ± 0.13	4.96 ± 0.14	5.10 ± 0.29	5.31 ± 0.32	4.95 ± 0.14	5.34 ± 0.30	5.47 ± 0.34	5.25 ± 0.23

Data are *n* (%) and mean ± SD

European, MixESA and SA groups are age and sex matched (4:1:4). Numbers of Europeans differ between Tables 1 and 2 as a consequence of different matching ratios

^a^Includes known (algorithmically defined) diabetes and HbA_1c_ > 47 mmol/mol (6.4%)

BMI was elevated in AC (29.6 kg/m^2^) compared with Europeans (27.1 kg/m^2^) (Tables 3 and 4). BMI in both second-generation AC (29.1 kg/m^2^) and MixEAC (28.0 kg/m^2^) was intermediate between first-generation AC and Europeans. Residence in the most deprived quintile was about threefold higher in AC (63%) vs Europeans (20%), and remained markedly elevated in second-generation migrants and MixEAC. Ever-smoking rates were lower in AC than in Europeans, but similar in second-generation migrants, and greater in MixEAC than in Europeans. Additional descriptive baseline characteristics by ethnic group are shown in ESM Table 1.

**Table 3:** Characteristics of UKB participants by ethnicity: European and AC origin groups by generation

Characteristic	European			AC second generation			AC first generation		
	All	Men	Women	All	Men	Women	All	Men	Women
*n* (%)	4400	1886 (43)	2514 (57)	2200	943 (43)	1257 (57)	2200	943 (43)	1257 (57)
Age, years	47.7 ± 5.8	47.3 ± 5.8	48.0 ± 5.8	47.5 ± 5.7	47.0 ± 5.6	47.8 ± 5.7	47.7 ± 5.8	47.3 ± 5.7	48.1 ± 5.9
Ever smoked	1322 (30)	598 (32)	724 (29)	655 (30)	329 (35)	326 (26)	344 (16)	223 (24)	121 (10)
Most deprived Townsend quintile	905 (21)	406 (22)	499 (20)	1323 (60)	554 (59)	769 (61)	1525 (69)	665 (71)	860 (68)
Years of education derived	15.9 ± 0.6	15.9 ± 0.5	15.9 ± 0.7	15.3 ± 0.9	14.8 ± 0.8	15.6 ± 0.7	15.9 ± 0.7	16.2 ± 0.7	15.8 ± 0.7
WHR	0.85 ± 0.09	0.92 ± 0.06	0.81 ± 0.07	0.86 ± 0.08	0.90 ± 0.07	0.84 ± 0.07	0.87 ± 0.07	0.91 ± 0.06	0.84 ± 0.07
Height, cm	169.5 ± 9.4	177.4 ± 6.8	163.6 ± 6.2	169.2 ± 8.9	176.2 ± 6.8	164 ± 6.3	167.4 ± 8.4	173.8 ± 6.6	162.6 ± 6.0
BMI, kg/m^2^	27.1 ± 5.0	27.7 ± 4.3	26.7 ± 5.5	29.1 ± 5.6	28.5 ± 4.7	29.6 ± 6.1	29.7 ± 5.4	28.2 ± 4.1	30.8 ± 5.9
Fat, %	30.5 ± 8.8	24.0 ± 5.8	35.3 ± 7.3	32.3 ± 9.6	24.3 ± 5.9	38.3 ± 7.2	33.9 ± 9.5	25.4 ± 5.4	40.3 ± 6.4
Birthweight, kg	3.3 ± 0.6	3.4 ± 0.6	3.3 ± 0.6	3.2 ± 0.7	3.4 ± 0.7	3.1 ± 0.6	3.4 ± 0.7	3.5 ± 0.7	3.3 ± 0.7
Known type 2 diabetes	112 (3)	69 (4)	43 (2)	113 (5)	57 (6)	56 (4)	184 (8)	86 (9)	98 (8)
All type 2 diabetes^a^	150 (3)	92 (5)	58 (2)	163 (7)	85 (9)	78 (6)	227 (10)	110 (12)	117 (9)
HbA_1c_, mmol/mol	34.5 ± 1.4	35.1 ± 0.7	34.0 ± 1.6	38.2 ± 2.5	39.0 ± 1.9	37.5 ± 2.8	39.1 ± 2.3	39.8 ± 1.9	38.4 ± 2.5
HbA_1c_, %	5.3 ± 2.3	5.4 ± 2.2	5.3 ± 2.3	5.5 ± 2.4	5.7 ± 2.3	5.6 ± 2.4	5.7 ± 2.4	5.8 ± 2.3	5.7 ± 2.4
Glucose, mmol/l	4.97 ± 0.16	5.06 ± 0.12	4.89 ± 0.15	5.18 ± 0.27	5.29 ± 0.21	5.08 ± 0.29	5.23 ± 0.32	5.26 ± 0.31	5.21 ± 0.33

Data are *n* (%) and mean ± SD

First/second generation assigned by year of migration. European and AC first- and second-generation groups are age and sex matched (2:1:1). Numbers of Europeans differ between Tables 3 and 4 as a consequence of different matching ratios

^a^Includes known (algorithmically defined) diabetes and HbA_1c_ > 47 mmol/mol (6.4%)

**Table 4:** Characteristics of UKB participants by ethnicity: European and AC origin groups by admixture

Characteristic	European			MixEAC			AC		
	All	Men	Women	All	Men	Women	All	Men	Women
*n* (%)	4180	1436 (34.4)	2744 (65.6)	1045	359 (34.4)	686 (65.6)	4180	1436 (34.4)	2744 (65.6)
Mean AC admixture, %	0.2 ± 1.1	0.2 ± 1.0	0.2 ± 1.1	43.1 ± 15.0	42.7 ± 15.4	43.3 ± 4.8	91.1 ± 11.3	90.8 ± 12.2	91.2 ± 10.8
Age, years	51.1 ± 7.8	51.2 ± 8.0	51.1 ± 7.6	50.9 ± 7.8	51.0 ± 8.0	50.8 ± 7.7	51.0 ± 7.8	51.1 ± 8.1	50.9 ± 7.6
Ever smoked	1289 (31)	515 (36)	774 (28)	439 (42)	160 (45)	279 (41)	844 (20)	412 (29)	432 (16)
Most deprived Townsend quintile	829 (20)	296 (21)	533 (19)	498 (48)	161 (45)	337 (49)	2634 (63)	906 (63)	1728 (63)
Years of education derived	15.5 ± 0.9	15.7 ± 0.5	15.5 ± 1.0	15.3 ± 0.8	15.2 ± 1.1	15.4 ± 0.7	15.4 ± 0.8	15.4 ± 1.1	15.5 ± 0.7
WHR	0.85 ± 0.09	0.93 ± 0.06	0.81 ± 0.07	0.85 ± 0.09	0.92 ± 0.07	0.82 ± 0.08	0.87 ± 0.08	0.91 ± 0.07	0.84 ± 0.07
Height, cm	168 ± 9	176 ± 7	164 ± 6	168 ± 9	177 ± 7	163 ± 7	167 ± 9	174 ± 7	163 ± 6
BMI, kg/m^2^	27.1 ± 5.0	27.8 ± 4.2	26.8 ± 5.3	28.0 ± 5.3	28.0 ± 4.3	28.0 ± 5.8	29.6 ± 5.5	28.3 ± 4.3	30.3 ± 5.9
Fat, %	32 ± 9	24.4 ± 5.9	35.6 ± 7.3	32.9 ± 9.2	24.5 ± 5.9	37.3 ± 7.4	34.7 ± 9.4	25.3 ± 5.8	39.6 ± 6.8
Birthweight, kg	3.3 ± 0.6	3.4 ± 0.7	3.3 ± 0.6	3.2 ± 0.7	3.3 ± 0.7	3.2 ± 0.7	3.3 ± 0.7	3.4 ± 0.7	3.2 ± 0.7
Known type 2 diabetes	109 (3)	51 (4)	58 (2)	51 (5)	22 (6)	29 (4)	416 (10)	183 (13)	233 (8)
All type 2 diabetes^a^	144 (3)	65 (5)	79 (3)	70 (7)	26 (7)	44 (6)	534 (13)	227 (16)	307 (11)
HbA_1c_, mmol/mol	34.8 ± 1.4	35.1 ± 1.2	34.6 ± 1.4	36.5 ± 2.1	37.2 ± 1.6	36.2 ± 2.3	39.2 ± 2.2	40.0 ± 2.2	38.7 ± 2.1
HbA_1c_, %	5.3 ± 2.3	5.4 ± 2.3	5.3 ± 2.3	5.5 ± 2.3	5.6 ± 2.3	5.5 ± 2.4	5.7 ± 2.4	5.8 ± 2.4	5.7 ± 2.3
Glucose, mmol/l	4.98 ± 0.14	5.04 ± 0.09	4.95 ± 0.15	4.95 ± 0.16	4.97 ± 0.17	4.94 ± 0.15	5.10 ± 0.25	5.23 ± 0.28	5.04 ± 0.20

Data are *n* (%) and mean ± SD

European, MixEAC and AC groups are age and sex matched (4:1:4). Numbers of Europeans differ between Tables 3 and 4 as a consequence of different matching ratios

^a^Includes known (algorithmically defined) diabetes and HbA_1c_ > 47 mmol/mol (6.4%)

### Multivariable and mediation analysis

**Fig. 3:**
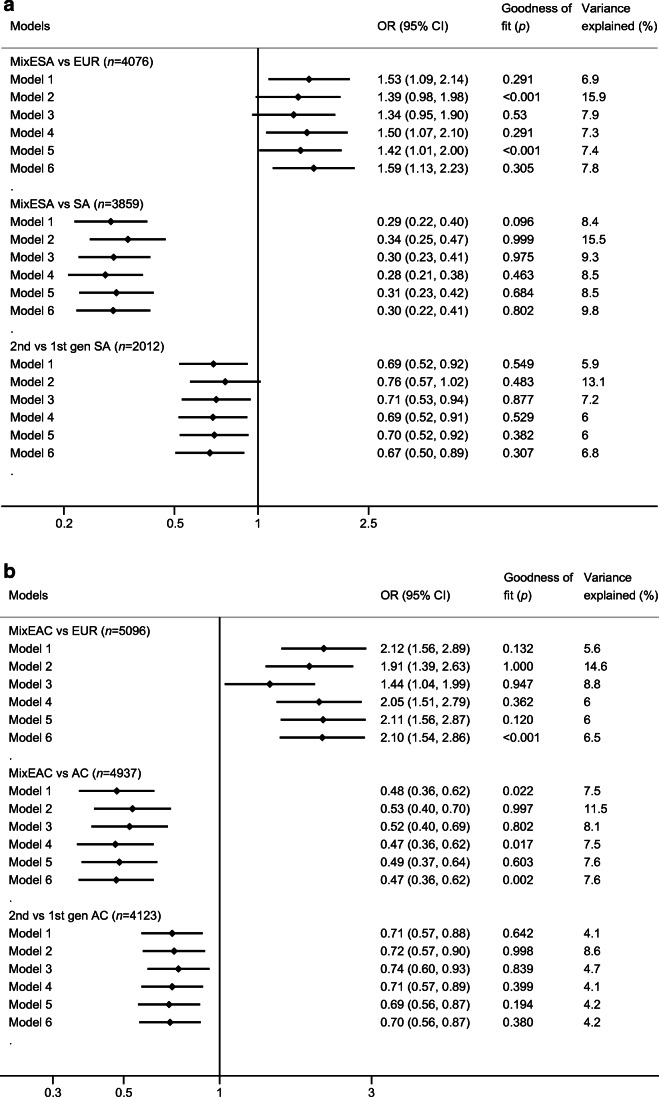
Forest plot of multivariable analysis for type 2 diabetes mellitus in SA (**a**) and AC (**b**). Model 1: Ethnicity/generations + age + sex; Model 2: Model 1 + WHR; Model 3: Model 1 + deprivation; Model 4: Model 1 + smoking; Model 5: Model 1 + height; Model 6: Model 1 + years of education. EUR, Europeans; gen, generation. Logarithmic base 10 scale (*x*-axis) shown

Measures of obesity and of SES made the strongest contribution in accounting for ethnic differences in type 2 diabetes (Fig. 3). The contributions of smoking, education and height were modest.

The relative contributions of these factors, individually and in combination, in mediating ethnic differences in risk depended on the comparison (Table 5). Thus, in MixESA, socioeconomic deprivation was the strongest mediator (accounting for 17%) of the excess risk of type 2 diabetes compared with Europeans (Table 5, ESM Figs 11,12). In contrast, the lower WHR in MixESA contributed most (15%) in mediating the lower risk of type 2 diabetes compared with SA (ESM Fig. 13). A similar pattern for WHR was observed for second- vs first-generation SA migrants. Similarly, in MixEAC, socioeconomic deprivation mediated the greatest amount (42%) of the excess risk of type 2 diabetes compared with Europeans, while the lower BMI in MixEAC mediated a greater amount (16%) of the lower risk of type 2 diabetes compared with AC. Deprivation and obesity contributed equally in mediating the lower risk of type 2 diabetes in second- vs first-generation AC migrants (ESM Fig. 14). In the SA analysis, about a fifth to a third of ethnic differences in type 2 diabetes prevalence appeared mediated by key environmental risk factors, alone or in combination (Table 5). In contrast, nearly two-thirds of the excess risk of type 2 diabetes in MixEAC vs Europeans was mediated by these environmental risk factors.

**Table 5:** Proportion of type 2 diabetes risk mediated by individual and joint effects of environmental risk factors comparing between generations of ethnic groups, between those of mixed and non-mixed ethnicity, and across genetic admixture

Variable	Total effect	Direct effect	% Mediated by									
			Smoking	Deprivation	WHR/BMI^b^	Education	Deprivation Smoking	Deprivation WHR/BMI^b^	Height WHR/BMI^b^	Education WHR/BMI^b^	Deprivation Education	Total
SA												
2nd vs 1st generation SA	−0.07	−0.05	−1	7	30	−4	0	3	1	−2	1	34^a^
MixESA vs Europeans	0.08	0.06	1	17	9	−7	1	6	3	−3	2	28^a^
MixESA vs SA	−0.25	−0.20	−2	2	15	3	0	1	1	1	0	21
SA admixture	0.39	0.31	0	4	13	0	0	0	0	0	0	22^a^
AC												
2nd vs 1st generation AC	−0.10	−0.08	0	11	11	−3	0	2	1	0	0	22
MixEAC vs Europeans	0.14	0.06	1	42	11	−1	1	5	0	−1	2	61^a^
MixEAC vs AC	−0.15	−0.11	1	9	16	0	0	2	1	0	0	29
AC admixture	0.35	0.23	0	18	12	0	0	6	0	0	0	35^a^

Total and direct effects are presented as log odds ratios (age and sex adjusted). The mediated percentages shown are rounded to the nearest integer

^a^Low contribution pathways have been omitted for the purpose of simplification. For these reasons the percentages might not add up to the total mediated

^b^For adiposity was used: WHR in the SA analyses, and BMI in the AC analyses

### Genetic admixture analysis

**Fig. 4:**
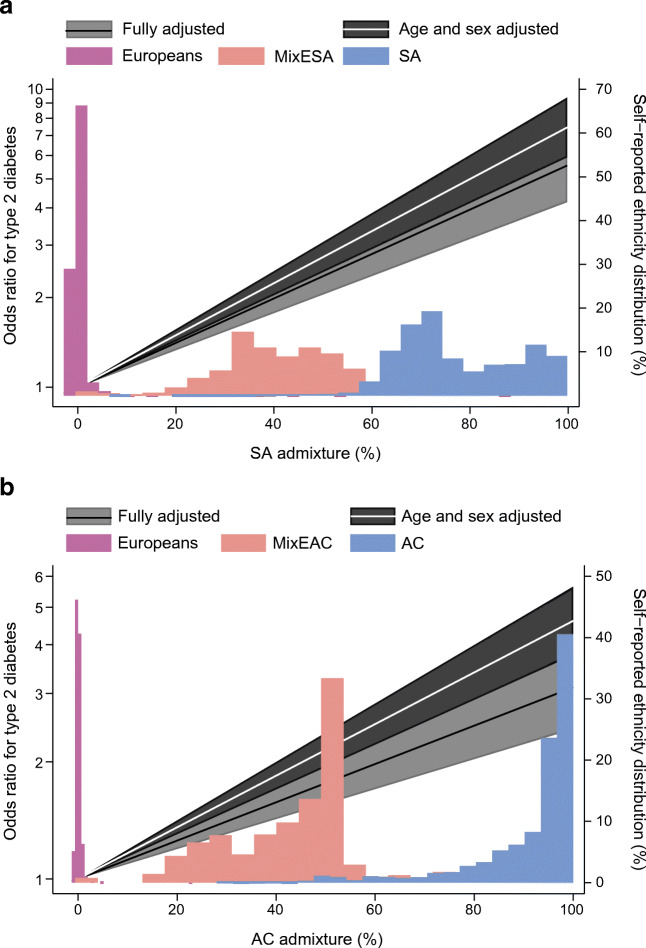
Odds ratios of type 2 diabetes and self-reported ethnicity distribution by SA (**a**) and AC (**b**) genetically assigned admixture. Odds ratios were based on fractional polynomials of power (1; linear). Fully adjusted for age, sex, smoking, deprivation, WHR for SA/BMI for AC, height and years of education; the shaded areas represent 95% CIs of the models. Coloured bars represent frequency of individuals by self-reported ethnicity

Genetic admixture level, as an estimate of ancestral and geographical proximity, correlated strongly with self-reported ethnicity (ESM Fig. 15). SA admixture was 48% in MixESA (Table 2, ESM Fig. 16). AC admixture was 43% in MixEAC (Table 4, ESM Fig. 17). Increasing admixture was associated with increasing type 2 diabetes risk (Fig. 4), to a similar extent to that presented for self-reported ethnicity. Mediation analysis, now using genetic admixture as the exposure, corroborated analyses for self-reported ethnicity, with environmental factors mediating 22% and 35% of excess type 2 diabetes in association with SA and AC admixture, respectively (Table 5, ESM Figs 11,14).

### Sensitivity analyses

Our initial DAG included physical activity and diet (Fig. 1, ESM Fig. 10). However, these behaviours were crudely assessed. The sensitivity analyses to determine the impact of physical activity and diet in accounting for ethnic differences in diabetes risk (ESM Fig.18) showed no additional impact of these measures, and these were dropped from subsequent mediation analysis. Similarly, although birthweight was considered an important mediator, only half of the sample had these data, severely diminishing analytical precision. Birthweight as a covariate did not impact on associations between ethnicity and diabetes risk (ESM Figs 19,20), and for this reason was also dropped from the final DAG (ESM Fig.10).

Repeating the multivariable analysis on ‘known’ type 2 diabetes instead of ‘all’, the effects were near identical (ESM Figs 21,22).

Replacing BMI for WHR in the SA analysis, and WHR for BMI in the AC analysis, accounted for a lower proportion of the observed difference in type 2 diabetes risk than the originally selected adiposity measure (ESM Figs 23,24).

Associations and mediation patterns were similar when HbA_1c_ replaced type 2 diabetes as the outcome (ESM Figs 25,26), and after excluding all those with diagnosed type 2 diabetes (ESM Table 2).

## Discussion

As expected, type 2 diabetes risks were substantially raised in SA and AC compared with European ethnic groups, but, importantly, we show that while second-generation migrants of SA and AC origin to the UK experience high rates of type 2 diabetes, these risks are 20% lower than in the first generation. We also found that type 2 diabetes risks in people of mixed ethnicity had risks closer to those of Europeans than to those of non-mixed ethnicity. Socioeconomic deprivation and measures of adiposity together accounted for about a fifth to a third of observed ethnic differences in type 2 diabetes risk. Finally, our findings suggest inter-generational changes in adiposity patterns may partially explain lower diabetes risks in subsequent generations.

Ethnic group membership is well established as a predictor of future diabetes, so we undertook mediation analysis to account for ethnic differences in prevalence in this cross-sectional analysis. The ~20% lower type 2 diabetes risk in second- vs first-generation migrants we found is similar to that achieved by lifestyle intervention on type 2 diabetes risk over 15 years in the Diabetes Prevention Programme [29]. We draw two conclusions from this comparison: first, that a 20% risk reduction is clinically important; and, second, that it is plausible that the observed magnitude of lower type 2 diabetes risk in second-generation migrants could be accounted for by inter-generational differences in environmental risk factors, including lifestyle. In mediation analyses, lower WHR in second-generation SA migrants appeared to account for a third of their lower risk of type 2 diabetes. A quarter of the lower type 2 diabetes risk in second- vs first-generation AC migrants was accounted for by a combination of SES and lower BMI. The impact of relatively modest inter-generational differences in adiposity measures (0.01 for WHR in SA, and 0.6 kg/m^2^ for BMI in AC) is striking. We performed a sensitivity analysis on the subsample with self-reported birthweight, to assess possible early life determinants of type 2 diabetes, and observed little impact, though we acknowledge the limitations of both self-reported birthweight and the reduced sample size. Acknowledging this imprecision, it is likely that environmental factors account for most of the lower type 2 diabetes risks in second-generation migrants. Our findings indicate the strong potential impact of environmental risk factor modification in addressing the higher rates of type 2 diabetes in these ethnic minority groups and confirm that these risks are not immutable.

In contrast to comparisons between first- and second-generation migrants, who differed mainly in terms of environmental exposures, when comparing mixed ethnic groups, both genetic backgrounds and environmental exposures were likely to differ. Type 2 diabetes risks in MixESA were about 70% lower than in SA, and about 50% lower in MixEAC than in AC. In both mixed populations, risks approached those of Europeans (1.5-fold excess in MixESA, and 2.0-fold excess in MixEAC). Known risk factors accounted for two-thirds of the excess risk of diabetes in MixEAC, but only one-third in MixESA. The main contributor in both mixed groups was SES; mixed ethnicity was associated with persistent socioeconomic disadvantage; in particular, 50% of MixEAC people resided in the poorest neighbourhoods, compared with 20% of Europeans. There was a smaller direct contribution from adiposity. Interestingly, the greater levels of education in MixESA mitigated somewhat against the potential excess risk of type 2 diabetes when compared with Europeans. In contrast, markedly lower adiposity levels in the mixed ethnicity samples, approaching those of Europeans, played a greater part in accounting for the 70% lower risk of type 2 diabetes in MixESA vs SA, and for the halving of risk of type 2 diabetes in MixEAC vs AC.

Using genetic admixture analysis, we found that clustering individuals by genetic similarity was strongly correlated with self-reported ethnicity. Deprivation and adiposity accounted for a third of the association between African admixture and type 2 diabetes, whereas WHR alone accounted for 13% of the association between SA admixture and type 2 diabetes. The association between genetic admixture and diabetes risk, once environmental risk factors were accounted for, approached linearity. A similar association has been previously observed for African admixture [13]. The percentage of African ancestry in self-assigned African Americans in those USA studies ranged from 78% to 85% [13, 16, 30]. While admixture panels differ, within UKB, AC have ~91% African ancestry, and those of MixEAC descent ~43%. Previous studies for African ancestry and type 2 diabetes, all from the USA, report that environmental factors, largely SES and obesity, account for one-third to two-thirds of the excess risk in African Americans [13, 16, 31].

We are likely to have underestimated the environmental risk factor contribution in accounting for ethnic differences in risk. We do not have longitudinal measures of factors such as obesity, and our measures are somewhat imprecise; we could not, for example, account for ectopic adiposity depots in the liver and elsewhere. Area of residence deprivation index cannot capture the entirety of individual socioeconomic disadvantage. Not all of the adverse effects of poor diet and lack of exercise will be captured by current adiposity status, and these former exposures were assessed by self-report only in the whole cohort. While reverse causality cannot operate for our main exposure (i.e. type 2 diabetes does not alter ethnic group membership), we acknowledge that this may affect mediation analyses, if, for example, the diagnosis of type 2 diabetes caused changes in health behaviours to reduce obesity. However, we also show similar associations when HbA_1c_, measured in all participants at recruitment, was employed as the outcome in sensitivity analyses.

We cannot exclude a contribution from genetic factors that both influence biology and correlate strongly with ethnic origin. But while ethnic-specific genetic variants for hyperglycaemia/diabetes have been reported [32–34] and different effects of known variants observed [35], these are insufficient to account for the observed marked ethnic differences in diabetes risk. It could be that variants that account for more upstream determinants of diabetes, such as adiposity measures, should be explored. Genetic determinants of body fat distribution have been described and may have differing and complex relations with diabetes risk. For example, a previous report has suggested that alleles predisposing to overall adiposity are surprisingly associated with lower risk of type 2 diabetes; individuals possessing these ‘favourable’ alleles were characterised by higher body fat percentage and higher subcutaneous fat, but lower liver fat and lower visceral/subcutaneous adipose tissue ratio [36]. In addition, recent diabetes remissions trials suggest that ectopic fat gain in organs such as the liver are likely causal for type 2 diabetes [37]. Taken together, these findings suggest that genetic determinants of adiposity may have divergent effects on risk of diabetes, possibly depending on the pattern of fat deposition. Whether differences in ‘favourable’ or ‘unfavourable’ adiposity alleles contribute to ethnic differences in diabetes risk is unknown.

There are limitations to this analysis. Response rates in UKB were <5%. Responders were likely to be healthier and of higher SES than non-responders, although this bias may differ by ethnicity. However, ethnic differences in diabetes prevalence in our study accord with those of previous, representative population cohorts [2]. The ethnic differences in socioeconomic deprivation observed in UKB reflect differentials observed from the census [38], though in some cases, notably for AC, are more marked in UKB. This in part reflects the older age of the UKB population. The census includes the whole population; the ethnic differential in SES is markedly attenuated in younger people, who for ethnic minority groups make up two-thirds of the population. Thus, comparisons including people of AC descent may have somewhat overestimated the contribution of socioeconomic deprivation in accounting for differences in type 2 diabetes. This does not undermine the conclusion of the study, that known risk factors, such as SES, make an important contribution to ethnic differences in diabetes risk. UKB is not appropriate for generalisable disease prevalence derivation. However, its plethora and heterogeneity of exposures and the large size could grant scientific inferences between exposures and health conditions that are generalisable to other populations [39], and the prevalence rates presented in this study were for comparison purposes only. Although separate European comparator groups were used due to the different age and sex distributions of the studied ethnic minority groups, they were all in separate analyses. We acknowledge the possibility of false discovery due to multiple comparisons among some of the same participants; however, there was a very low degree of overlap and we have interpreted these data cautiously. The first- and second-generation migrants in this study are a mix of families and unrelated individuals. Unfortunately, neither kinship data nor data on family history of type 2 diabetes were collected, so it was not possible to explore these potential sources of bias. Years of education were derived from qualification(s) using the ISCED coding, which may not be comparable across countries due to differential access to educational opportunities, particularly for women. While UKB is large, it includes relatively few people of mixed ethnicity, and the numbers of those of ethnic minority groups who could readily be matched by age and sex to people of mixed ethnicity were modest, limiting the power of the analysis. Further research should include replication in other datasets. However, the inclusion of those of mixed ethnicity is unique, and performing a mixed ethnicity and inter-generational analysis, using self-reported and genetic ancestry to assign ethnicity, is a strength, as it enables the employment of different approaches to address the same question.

### Conclusions

The excess diabetes risk seen in first-generation SA and AC migrants compared with Europeans persists in the second generation and is also observed in those of mixed ethnicity. Known risk factors accounted for a greater proportion of the ethnic differences in diabetes in those of AC vs SA heritage. Continued social disadvantage makes a strong contribution to that part of the excess risk of diabetes that we could account for. Importantly, however, we also show that even slightly better social circumstances and adiposity are associated with a lower risk of type 2 diabetes, and, given these two factors are alterable, we conclude that people from minority ethnic groups are not necessarily destined to suffer from a greater burden of diabetes.

## Supplementary Information

ESM (PDF 5.09 MB)

## Data Availability

UK Biobank data are available through a procedure described at www.ukbiobank.ac.uk/using-the-resource/. This research has been conducted using the UK Biobank Resource Application ID 7661.

## Abbreviations

AC: African Caribbean
DAG: Directed acyclic graph
ISCED: International Standard Classification of Education
MixEAC: Mixed European/African Caribbean
MixESA: Mixed European/South Asian
PC: Principal component
SA: South Asian
SES: Socioeconomic status
UKB: UK Biobank
WHR: Waist/hip ratio
