# Supplementary material for: Type 2 diabetes risks and determinants in second-generation migrants and mixed ethnicity people of South Asian and African Caribbean descent in the UK
Source: Diabetologia. 2021 Oct 20;65(1):113–27. doi: 10.1007/s00125-021-05580-7 (PMC8660755; doi:10.1007/s00125-021-05580-7)
Supplement: Supplementary file 1 — (PDF 5.09 MB) [file 125_2021_5580_MOESM1_ESM.pdf]

Electronic supplementary material (ESM)

METHODS

Dietary patterns

All frequencies were quantified on a weekly basis and similar foods incorporated into food groups – fruits: dried and fresh, vegetables: salad, cooked and raw, fish: oily and non-oily, red meat: beef, lamb, mutton and pork.

The Kaiser–Meyer–Olkin test of sphericity and Barlett's criterion was 0·63, implying high interrelationships between food variables. The orthogonal varimax rotation was used to derive optimal non-correlated components (dietary patterns) and we decided to retain two components. Based on the fact that factor loadings/correlation coefficients represent the correlation of each predicting variable with the dietary pattern score, higher absolute values indicate that the variable contributes more to the construction of this particular pattern. The dietary patterns were named according to scores of the predicting variables that correlated most with the component/pattern. The two main components emerging from the PCA were a 'healthy' dietary pattern (component 1) that was characterised by fruit, vegetable, fish and water consumption, and an 'unhealthy' diet variable which included red meat, processed meat and coffee drinking. In order to assign each individual to one of the components we created tertiles of scores for healthy and unhealthy patterns. Individuals in the two highest tertiles of the healthy pattern (component 1) and in the lowest of unhealthy pattern (component 2) were grouped as following a healthy diet. Individuals in the two highest tertiles of the unhealthy pattern (component 2) and in the lowest healthy pattern (component 1) were grouped as following an unhealthy diet.

Genetic data quality control (QC)

38,598 participants remained in the non-European UK Biobank dataset after exclusion of gender mismatches, missingness/heterozygosity outliers, participants with excessive genetic relatedness, no QC metrics, individuals that had withdrawn their consent and European participants.

GENESIS R package provides statistical methodology for analysing genetic data from samples with population structure and/or familial relatedness. This analysis was based on a subset of 171,258 single nucleotide polymorphisms (SNP) of the non-European dataset (applying the same thresholds with UK Biobank PCA analysis (1), including missing rate > 0.015, minor allele frequency (MAF) <0.01, markers in regions of long-range linkage disequilibrium as provided by UK Biobank and pruning to a set of independent markers such that pairwise $r^2 < 0.1$, using windows of 1000 markers and a step-size of 80 markers). PCA was performed with the PC-AiR algorithm that accounts for known or cryptic relatedness, to obtain PCs that capture population, rather than family structure (2).

Using the more precise GENESIS principal components (PCs) we applied five k-means on the non-EUR sample in order to identify and remove individuals with Chinese ancestry (Figure 5.1). We then applied ten k-means (Figure 5.2) and retained nine clusters: five for the SA admixture analysis (clusters 1, 7, 8, 9, and 10) and five for the AC admixture analysis (clusters 2, 4, 5, 6, and 10), with cluster 10 in common. Cluster 3 was excluded as it included the most heterogeneous admixture between SA and AC.

RESULTS

ESM Table 1: Additional baseline characteristics of UK Biobank participants by ethnicity; European, South Asian an African Caribbean origin groups. Data are n (%) and mean (standard deviation). First/second-generation assigned by year of migration. European and South Asian/ African Caribbean first- and second-generation groups are age and sex-matched (2:1:1). European, Mixed and South Asian/ African Caribbean groups are age and sex-matched (4:1:4).

	**European**			**South Asian second generation**			**South Asian first generation**		
	**All**	**Males**	**Females**	**All**	**Males**	**Females**	**All**	**Males**	**Females**
**n (%)**	2230	1072 (48)	1158 (52)	1115	536 (48)	579 (52)	1115	536 (48)	579 (52)
**Age, yrs**	47.1±6.7	46.6±6.4	47.5±6.9	46.6±6.7	46.2±6.5	47.0±6.9	46.9±6.7	46.5±6.5	47.3±6.9
**Townsend index**	-1.19 (0.25)	-1.10 (0.22)	-1.28 (0.25)	0.32 (0.40)	0.36 (0.49)	0.29 (0.29)	0.62 (0.42)	0.96 (0.21)	0.31 (0.31)
**Physical activity >10 mins, days/ wk**	3.5±2.3	3.5±2.3	3.5±2.3	3.4±2.3	3.3±2.4	3.5±2.3	3.4±2.4	3.4±2.3	3.4±2.4
**Weight, kg**	79±17	87±15	71±15	75±16	82±15	69±14	73±14	80±13	68±13
**Fat mass, kg**	23.8±9.8	21.7±8.4	25.7±10.6	24.0±9.4	21.4±7.8	26.3±10.2	23.5±8.5	20.7±6.9	26.0±7.1
**Diet**									
**"Healthy", n (%)**	520 (23)	169 (16)	351 (30)	367 (33)	134 (25)	233 (40)	479 (43)	204 (38)	275 (48)
**"Unhealthy", n (%)**	582 (26)	364 (34)	218 (19)	134 (12)	90 (17)	44 (8)	81 (7)	44 (8)	37 (6)
	**European**			**Mixed European/ South Asian**			**South Asian**		
**n (%)**	3324	1392 (41.9)	1932 (58.1)	831	348 (41.9)	483 (58.1)	3317	1392(41.9)	1925(58.1)
**Age, yrs**	52.3±8.5	51.7±8.4	52.6±8.5	52.2±8.5	51.5±8.4	52.6±8.5	52.1±8.5	51.6±8.5	52.5±8.5
**Townsend index**	-1.47 (0.33)	-1.49 (0.32)	-1.45 (0.33)	-0.26 (0.37)	-0.15 (0.46)	-0.34 (0.26)	0.28 (0.37)	0.48 (0.39)	0.14 (0.28)
**Physical activity >10 mins, days/ wk**	3.6±2.3	3.6±2.3	3.5±2.4	3.5±2.4	3.5±2.4	3.6±2.4	3.5±2.4	3.4±2.3	3.6±2.4
**Weight, kg**	77.6±16.3	85.9±14.3	71.7±15.1	74.6±15.7	82.7±14.8	68.7±13.7	72.6±14.2	78.9±13.3	68.1±13.0
**Fat mass, kg**	25±10	22±8	27±11	24±10	21±9	26±10	24±9	21±7	27±9
**Diet**									
**"Healthy", n (%)**	785 (24)	208 (15)	577 (30)	240 (29)	68 (20)	172 (36)	1531 (46)	515 (37)	1016 (53)
**"Unhealthy", n (%)**	811 (24)	460 (33)	351 (18)	183 (22)	106 (31)	77 (16)	212 (6)	125 (9)	87 (5)
	**European**			**African Caribbean second generation**			**African Caribbean first generation**		
**n (%)**	4400	1886 (43)	2514 (57)	2200	943 (43)	1257 (57)	2200	943 (43)	1257 (57)
**Age, yrs**	47.7±5.8	47.3±5.8	48.0±5.8	47.5±5.7	47.0±5.6	47.8±5.7	47.7±5.8	47.3±5.7	48.1±5.9
**Townsend index**	-1.2 (0.27)	-1.12 (0.26)	-1.26 (0.26)	2.28 (0.27)	2.23 (0.19)	2.31 (0.32)	3.14 (0.48)	3.36 (0.47)	2.97 (0.41)
**Physical activity >10 mins, days/ wk**	3.5±2.3	3.5±2.3	3.5±2.3	3.6±2.3	3.6±2.3	3.6±2.3	3.5±2.3	3.5±2.3	3.5±2.2
**Weight, kg**	78±17	87±15	72±15	84±18	89±16	80±18	83±16	85±14	81±17

**Fat mass, kg**	24±10	22±8	26±11	28±12	22±9	32±13	29±12	22±8	34±12
**Diet**									
**"Healthy", n (%)**	1038 (24)	267 (14)	771 (31)	610 (28)	175 (19)	435 (35)	593 (27)	152 (16)	441 (35)
**"Unhealthy", n (%)**	1213 (28)	701 (37)	512 (20)	391 (18)	210 (22)	181 (14)	236 (11)	138 (15)	98 (8)

	**European**			**Mixed European/ African Caribbean**			**African Caribbean**		
**n (%)**	4180	1436(34.4)	2744(65.7)	1045	359(34.4)	686(65.7)	4180	1436(34.4)	2744(65.7)
**Age, yrs**	51.1±7.8	51.2±8.0	51.1±7.6	50.9±7.8	51.0±8.0	50.8±7.7	51.0±7.8	51.1±8.1	50.9±7.6
**Townsend index**	-1.31 (0.25)	-1.22 (0.31)	-1.36 (0.19)	1.13 (0.52)	0.88 (0.44)	1.27 (0.51)	2.60 (0.26)	2.77 (0.34)	2.52 (0.15)
**Physical activity >10 mins, days/ wk**	3.5±2.3	3.5±2.3	3.5±2.3	3.7±2.3	3.8±2.3	3.6±2.3	3.6±2.2	3.6±2.2	3.6±2.2
**Weight, kg**	76.8±16.2	86.514.3	71.7±14.7	79.0±16.5	87.4±14.6	74.7±15.8	82.216.1	86.0±14.9	80.2±16.3
**Fat mass, kg**	25±10	22±8	27±11	27±11	22±8	29±11	29±12	22±8	33±12
**Diet**									
**"Healthy", n (%)**	1024 (25)	213 (15)	811 (30)	297 (28)	58 (16)	239 (35)	1339 (32)	294 (21)	1045 (38)
**"Unhealthy", n (%)**	1052 (25)	504 (35)	548 (20)	239 (23)	117 (33)	122 (18)	455 (11)	210 (15)	245 (9)

ESM Table 2: Mediational model between ethnic groups and level of admixture and the association with All HbA1c levels (for comparison purposes) and after excluding those with "Known" type 2 diabetes, which is explained by five mediators (smoking, deprivation, BMI, height and years of education) and their interrelationships. The numbers are standardised estimates, age and sex adjusted. The mediated percentages shown are rounded to the nearest integer and for this reason they might not add up to the total (*). Abbreviations- WHR: waist to hip ratio, BMI: body mass index.

Ethnicity	Total effect	Direct effect	% Mediated by													
			Smoking	Deprivation	WHR/BMI	Education	Deprivation Smoking	Education Smoking	Deprivation WHR/BMI	Height WHR/BMI	Education WHR/BMI	Deprivation Education	Deprivation Education Smoking	Deprivation Height WHR/BMI	Deprivation Education WHR/BMI	Total
ESA vs EUR																
All HbA1c	0.095	0.083	1	8	4	-4	1	0	2	1	-1	1	0	0	0	13
HbA1c excluding diagnosed T2D	**0.097**	**0.090**	**0**	**5**	**2**	**-4**	**1**	**0**	**1**	**1**	**-1**	**1**	**0**	**0**	**0**	**7**
ESA vs SA																
All HbA1c	-0.153	-0.107	0	3	19	4	0	0	0	1	1	1	1	0	0	30
HbA1c excluding diagnosed T2D	**-0.140**	**-0.103**	**1**	**1**	**17**	**3**	**0**	**0**	**1**	**1**	**1**	**1**	**0**	**0**	**0**	**26**
SA admixture																
All HbA1c	0.285	0.225	0	5	11	0	0	0	2	2	0	2	0	0	0	21*
HbA1c excluding diagnosed T2D	**0.258**	**0.218**	**0**	**4**	**9**	**-1**	**0**	**0**	**1**	**2**	**0**	**1**	**0**	**0**	**0**	**16**
EAC vs EUR																
All HbA1c	0.117	0.072	2	22	8	-1	2	0	4	0	0	1	0	0	1	38*
HbA1c excluding diagnosed T2D	**0.123**	**0.090**	**2**	**14**	**6**	**0**	**2**	**0**	**2**	**0**	**0**	**1**	**0**	**0**	**0**	**27***
EAC vs AC																
All HbA1c	-0.108	-0.086	-4	4	17	0	0	0	2	1	0	0	0	0	0	20
HbA1c excluding diagnosed T2D	**-0.097**	**-0.075**	**-8**	**6**	**20**	**0**	**0**	**0**	**3**	**1**	**0**	**0**	**0**	**0**	**0**	**22**
AC admixture																
All HbA1c	0.251	0.193	-2	9	13	0	0	0	2	0	0	0	0	0	0	23*
HbA1c excluding diagnosed T2D	**0.249**	**0.192**	**-4**	**10**	**12**	**-1**	**1**	**0**	**3**	**0**	**0**	**1**	**0**	**0**	**0**	**23***

Matching procedure

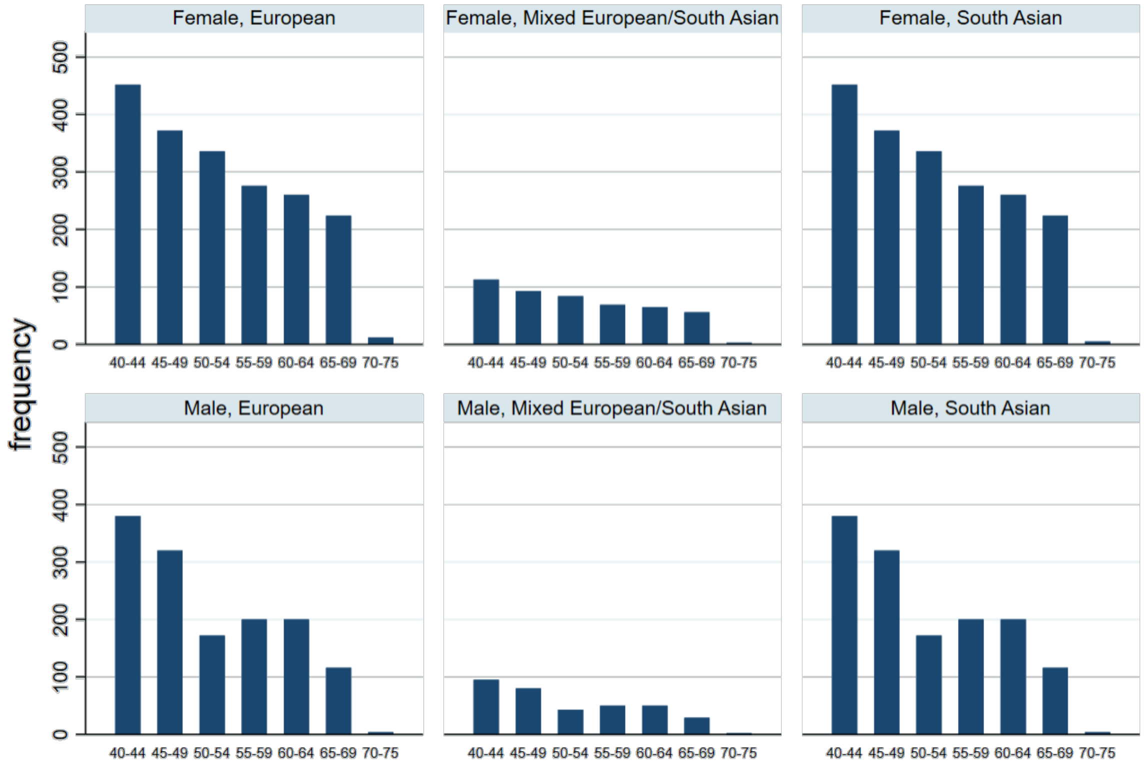

ESM Figure 1: Frequency distribution by age, sex and European/South Asian ethnicity. Mixed European/South Asians (ESA) (n=831) – South Asians (SA) – Europeans (1:4:4, N=7,479). The reference ethnic group is the mixed ethnicity group. Matching for each comparison was performed independently for sex and five-year age strata.

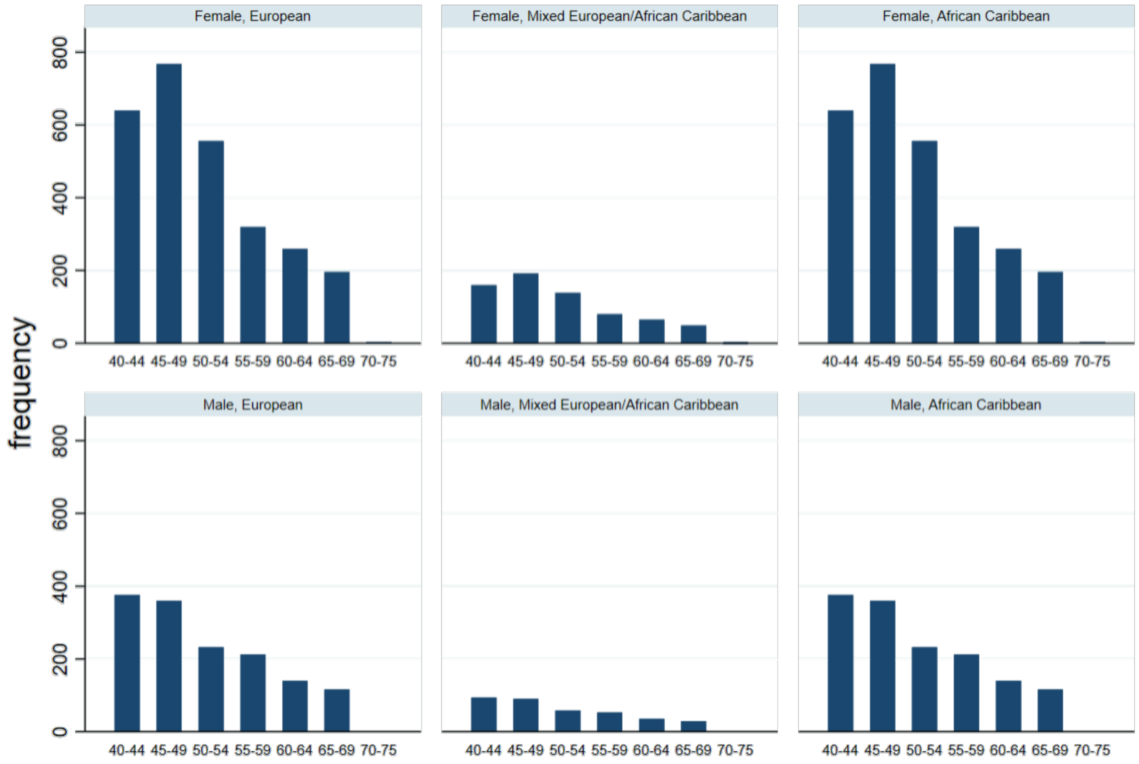

ESM Figure 2: Frequency distribution by age, sex and European/African Caribbean ethnicity. Mixed European/African Caribbeans (EAC) (n=1,045) – African Caribbeans (AC) – Europeans (1:4:4, N=9,405). The reference ethnic group is the mixed ethnicity group. Matching for each comparison was performed independently for sex and five-year age strata.

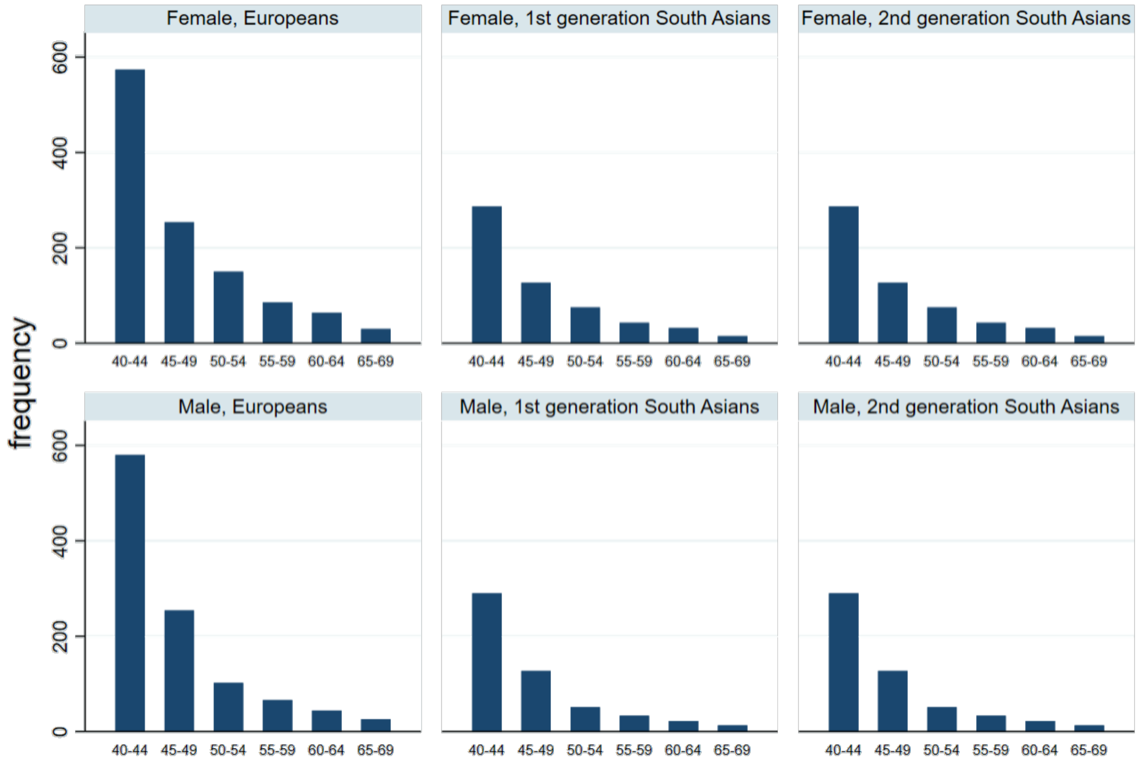

ESM Figure 3: Frequency distribution by age, sex and generations of South Asians. Second generation SA (n=1,115) – First generation SA – Europeans (1:1:2, N=4,460). The reference ethnic group is the second-generation group. Matching for each comparison was performed independently for sex and five-year age strata.

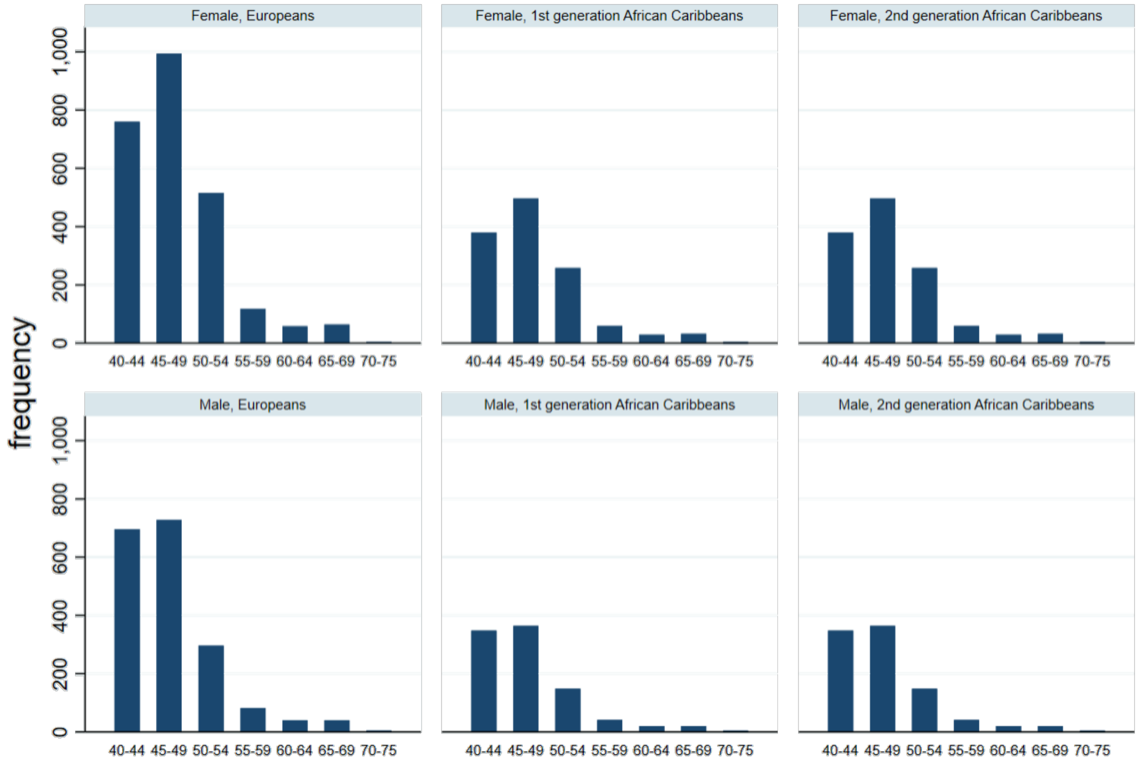

ESM Figure 4: Frequency distribution by age, sex and generations of African Caribbeans. Second generation AC (n=2,200) – First generation AC – Europeans (1:1:2, N=8,800). The reference ethnic group is the second-generation group. Matching for each comparison was performed independently for sex and five-year age strata.

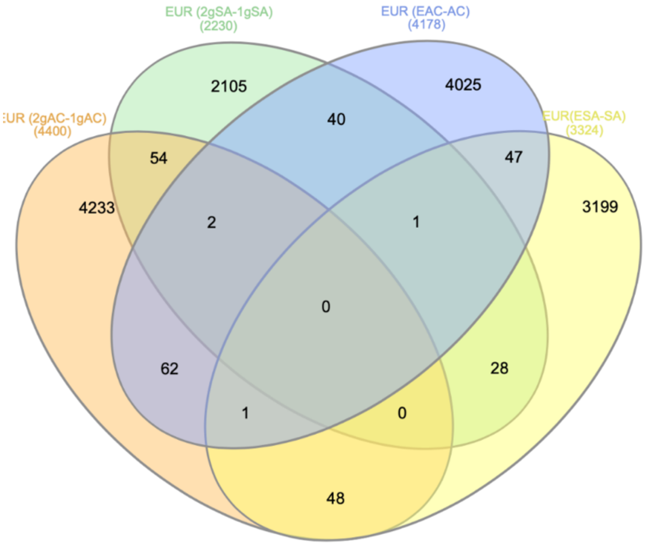

ESM Figure 5: Degree of overlap in the European comparator samples – Created with (3)

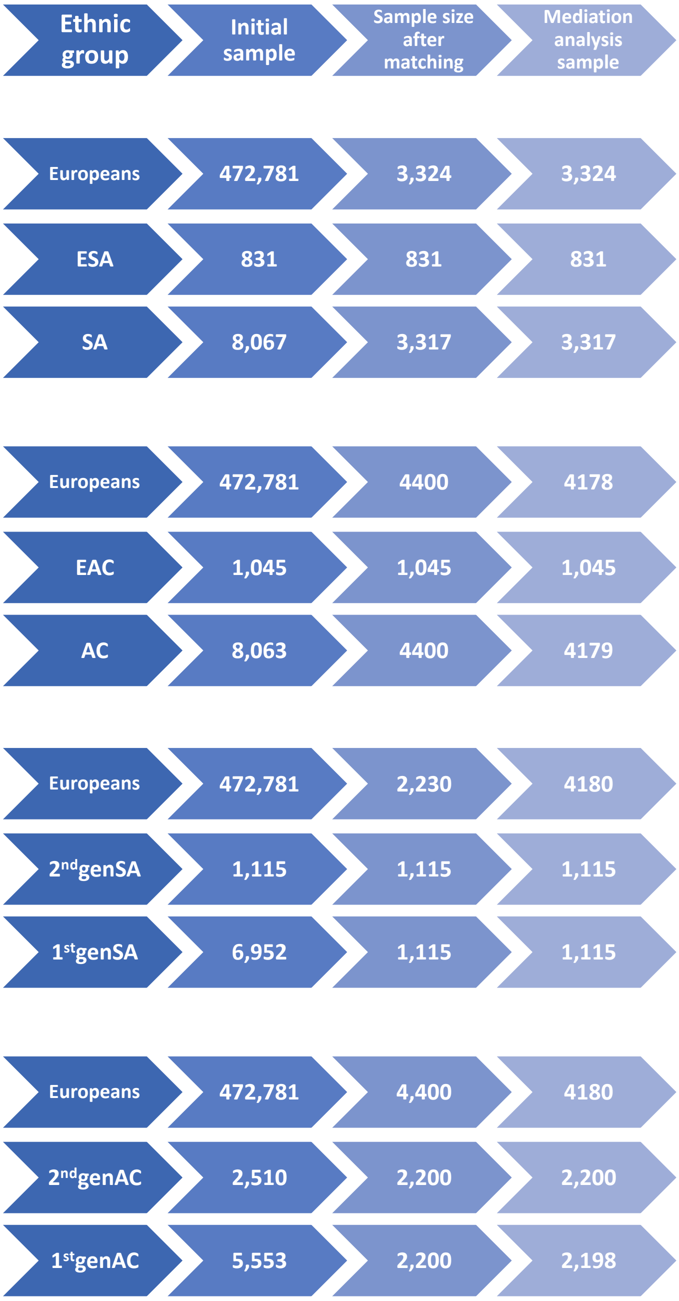

ESM Figure 6: Flow diagram of sample size in each stage by comparison group.

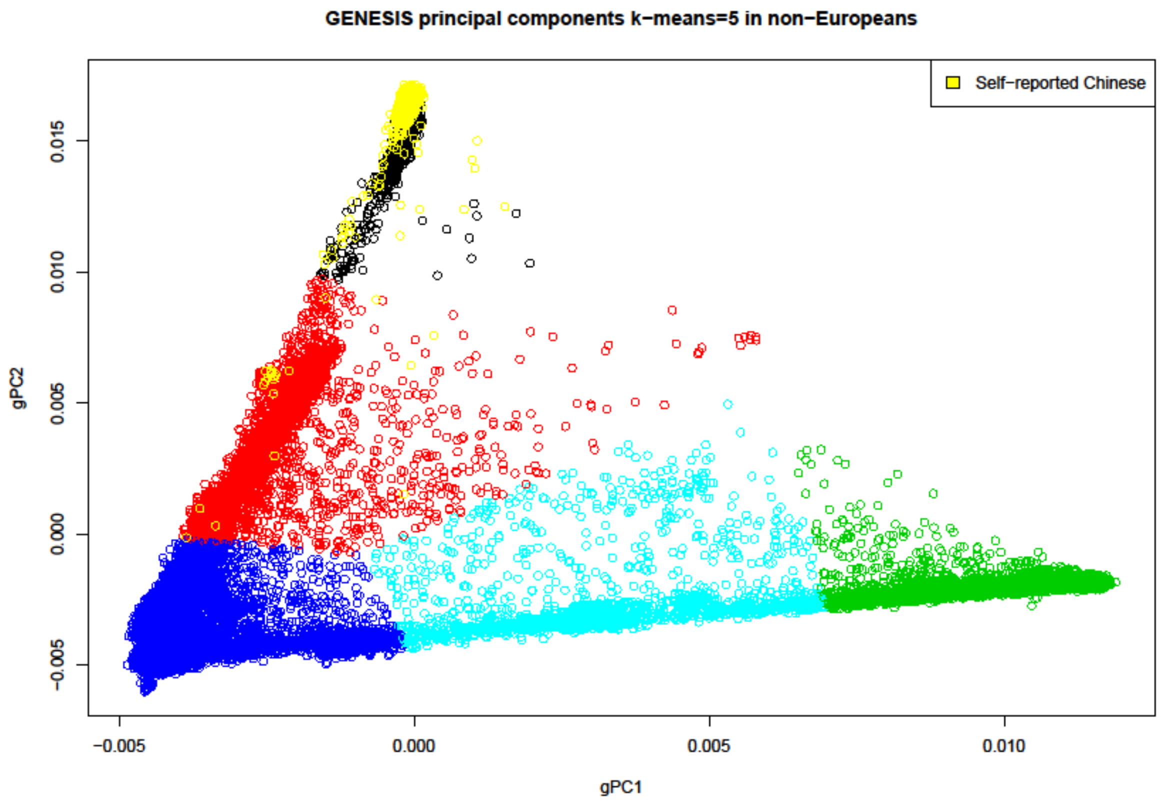

ESM Figure 7: GENESIS principal components (PCs) using five k-means on the non-EUR sample. We identified and removed individuals who were clustered in terms of Chinese ancestry alongside their self-reported ethnicity.

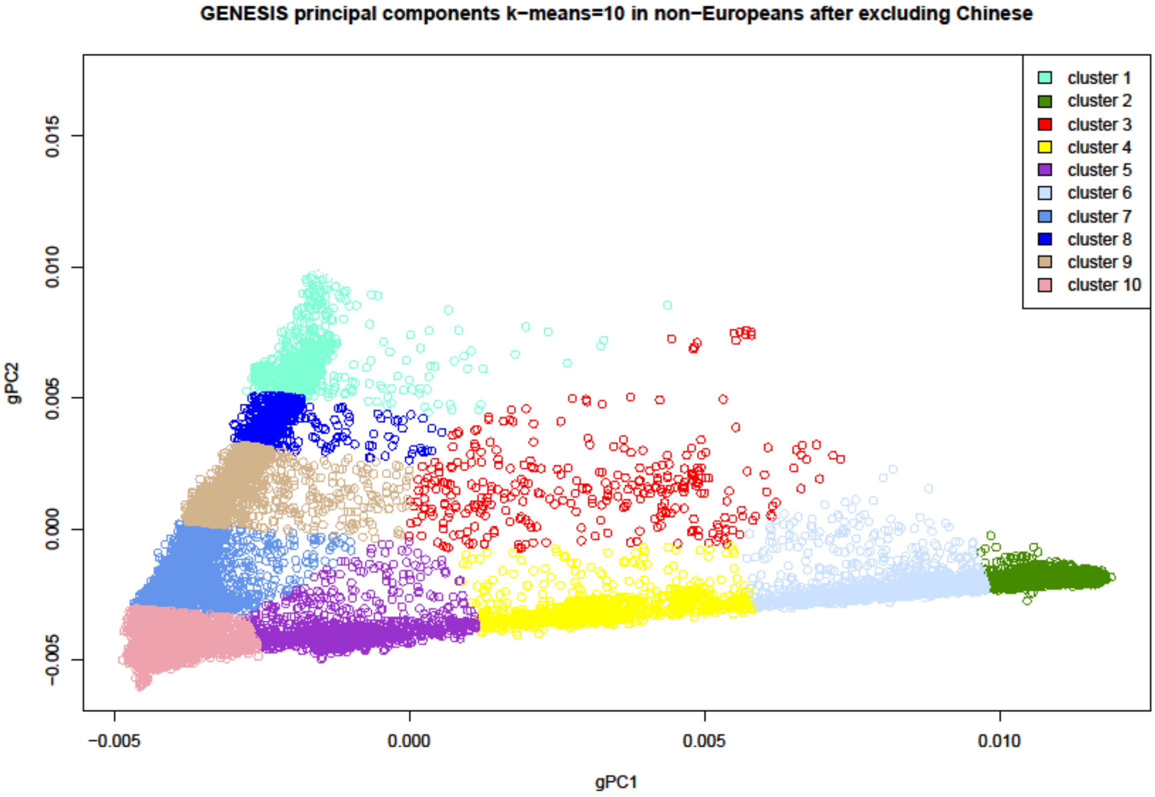

ESM Figure 8: GENESIS principal components (PCs) using ten k-means on the non-Europeans after excluding Chinese sample.

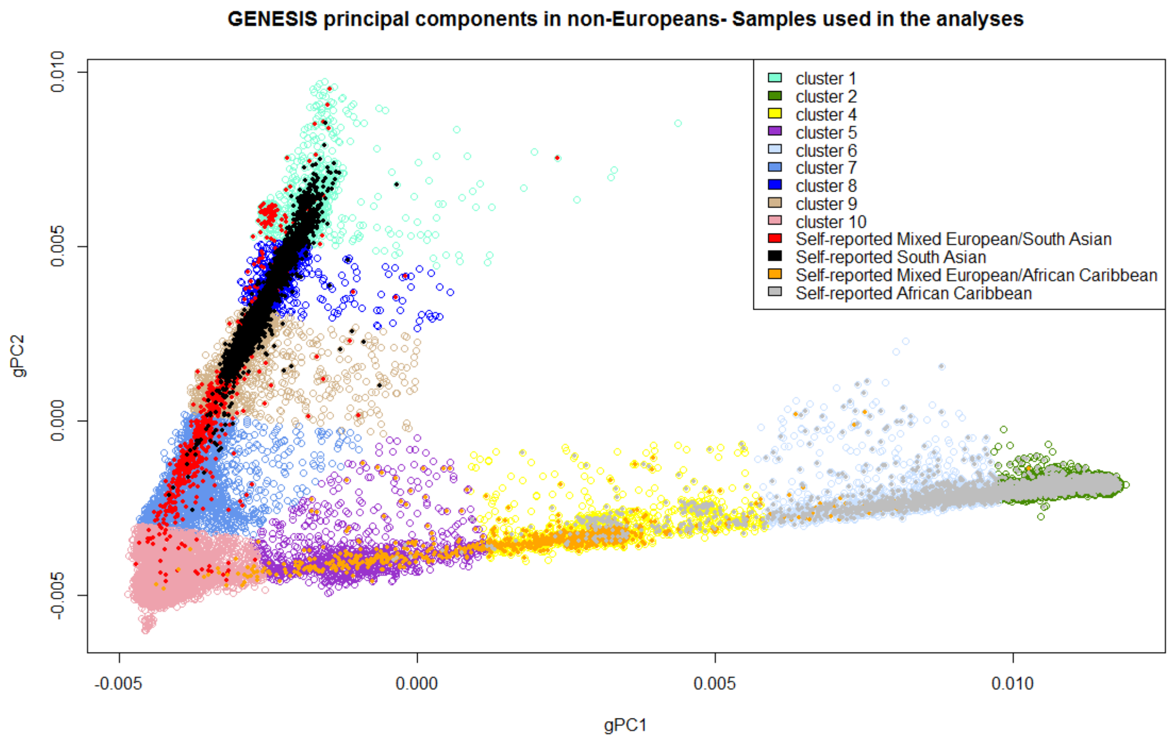

ESM Figure 9: GENESIS principal components (PCs) in the non-Europeans- samples used in our analyses.

Mediation path analysis

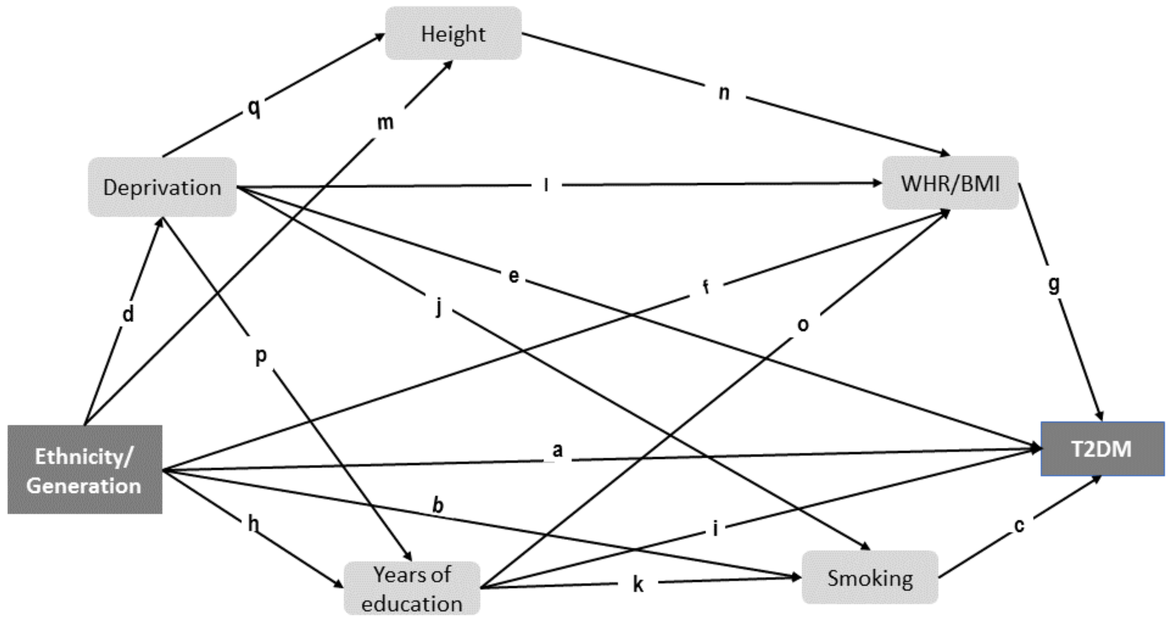

ESM Figure 10: Directed acyclic graph of ethnicity on type 2 diabetes in its finalised form for mediation analysis. Individual paths are labelled with lower case letters. Abbreviations-T2DM: type 2 diabetes mellitus.

A

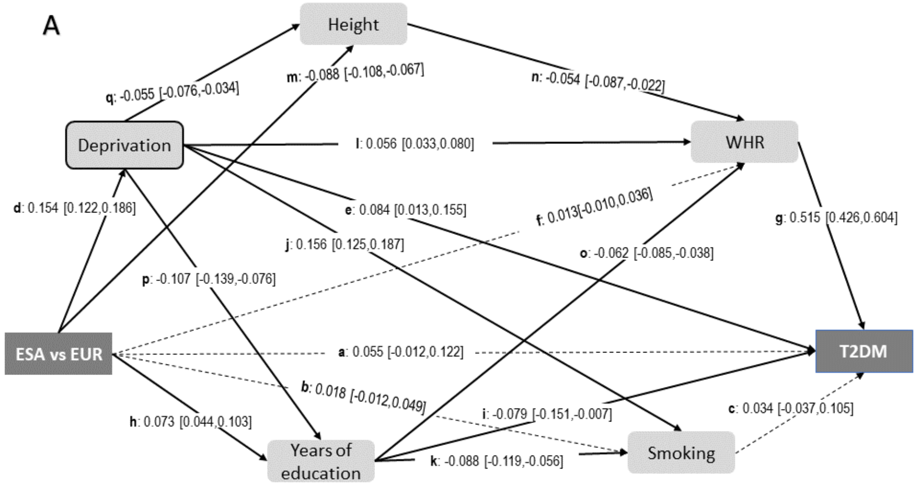

Ethnicity	Total effect	Direct effect	% Mediated by Smoking	Deprivation	WHR	Education	Deprivation Smoking	Education Smoking	Deprivation WHR	Height WHR	Education WHR	Deprivation Education	Deprivation Education Smoking	Deprivation Height WHR	Deprivation Education WHR	Total
Pathway		(a)	(b-c)	(d-e)	(f-g)	(h-i)	(d-h-c)	(i-j-c)	(d-l-g)	(m-n-g)	(h-o-g)	(d-p-i)	(d-p-k-c)	(d-q-n-g)	(d-p-o-g)	
ESA vs EUR	0.076	0.055	1	17	9	-7	1	0	6	3	-3	2	0	0	1	**28***

B

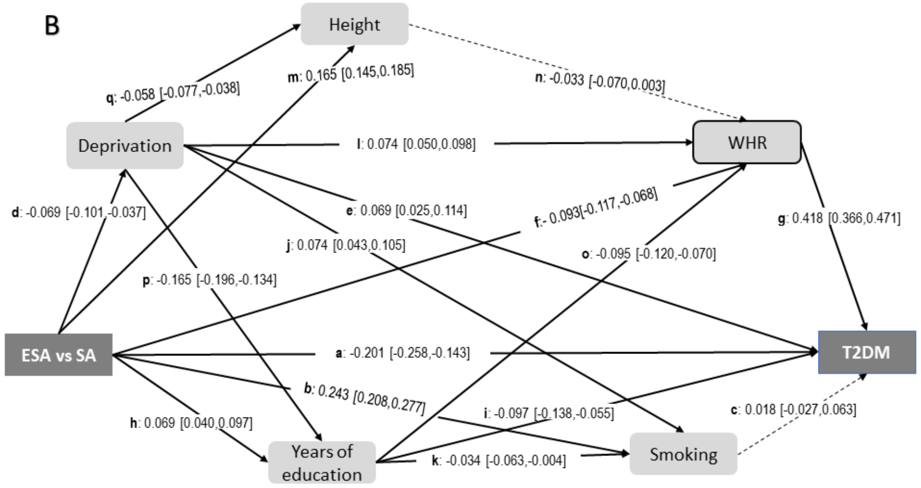

Ethnicity	Total effect	Direct effect	% Mediated by Smoking	Deprivation	WHR	Education	Deprivation Smoking	Education Smoking	Deprivation WHR	Height WHR	Education WHR	Deprivation Education	Deprivation Education Smoking	Deprivation Height WHR	Deprivation Education WHR	Total
Pathway		(a)	(b-c)	(d-e)	(f-g)	(h-i)	(d-h-c)	(i-j-c)	(d-l-g)	(m-n-g)	(h-o-g)	(d-p-i)	(d-p-k-c)	(d-q-n-g)	(d-p-o-g)	
ESA vs SA	-0.254	-0.201	-2	2	15	3	0	0	1	1	1	0	0	0	0	**21**

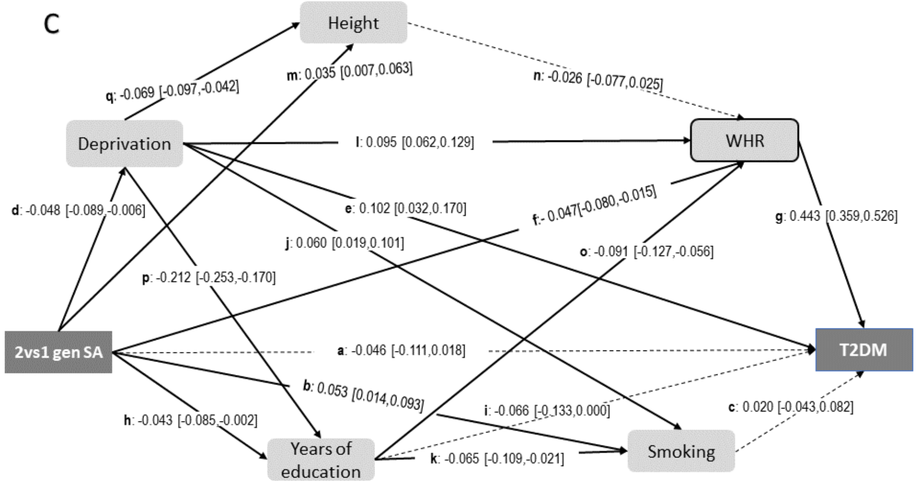

	Total effect	Direct effect	% Mediated by													
Generation			Smoking	Deprivation	WHR	Education	Deprivation Smoking	Education Smoking	Deprivation WHR	Height WHR	Education WHR	Deprivation Education	Deprivation Education Smoking	Deprivation Height WHR	Deprivation Education WHR	Total
Pathway		(a)	(b-c)	(d-e)	(f-g)	(h-i)	(d-h-c)	(i-j-c)	(d-l-g)	(m-n-g)	(h-o-g)	(d-p-i)	(d-p-k-c)	(d-q-n-g)	(d-p-o-g)	
2vs1 SA	-0.07	-0.046	-1	7	30	-4	0	0	3	1	-2	1	0	0	1	**34***

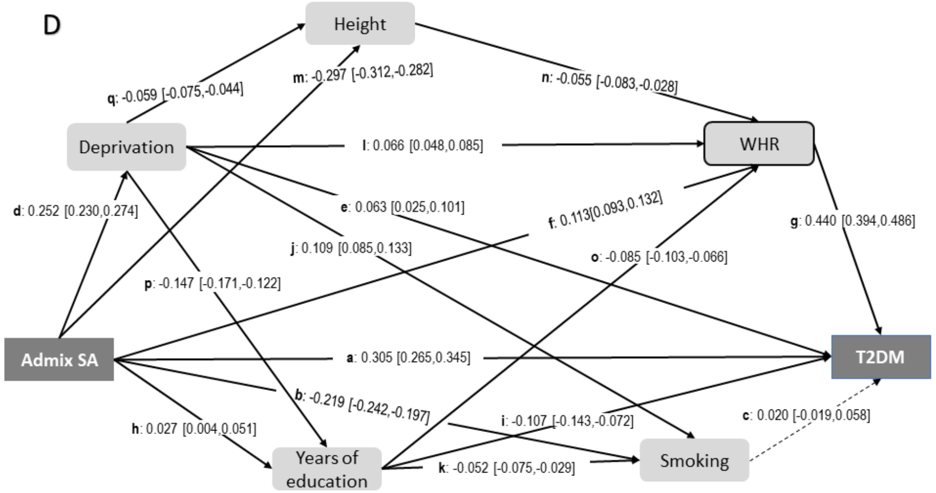

	Total effect	Direct effect	% Mediated by													
Admixture			Smoking	Deprivation	WHR	Education	Deprivation Smoking	Education Smoking	Deprivation WHR	Height WHR	Education WHR	Deprivation Education	Deprivation Education Smoking	Deprivation Height WHR	Deprivation Education WHR	Total
Pathway		(a)	(b-c)	(d-e)	(f-g)	(h-i)	(d-h-c)	(i-j-c)	(d-l-g)	(m-n-g)	(h-o-g)	(d-p-i)	(d-p-k-c)	(d-q-n-g)	(d-p-o-g)	
SA	0.391	0.305	0	4	13	0	0	0	0	0	0	0	0	0	0	**22***

ESM Figure 11: Diagrams of mediational model between South Asian ethnic groups (A, B), generations (C) and level of admixture (D) and the proportion of the observed difference in type 2 diabetes prevalence, which is explained by five mediators (smoking, deprivation, WHR, height, and years of education) and their interrelationships. The dashed arrows indicate non-significant association and the numbers are standardised estimates, age and sex adjusted. The mediated percentages shown are rounded to the nearest integer and for this reason they might not be added up to the total (*).

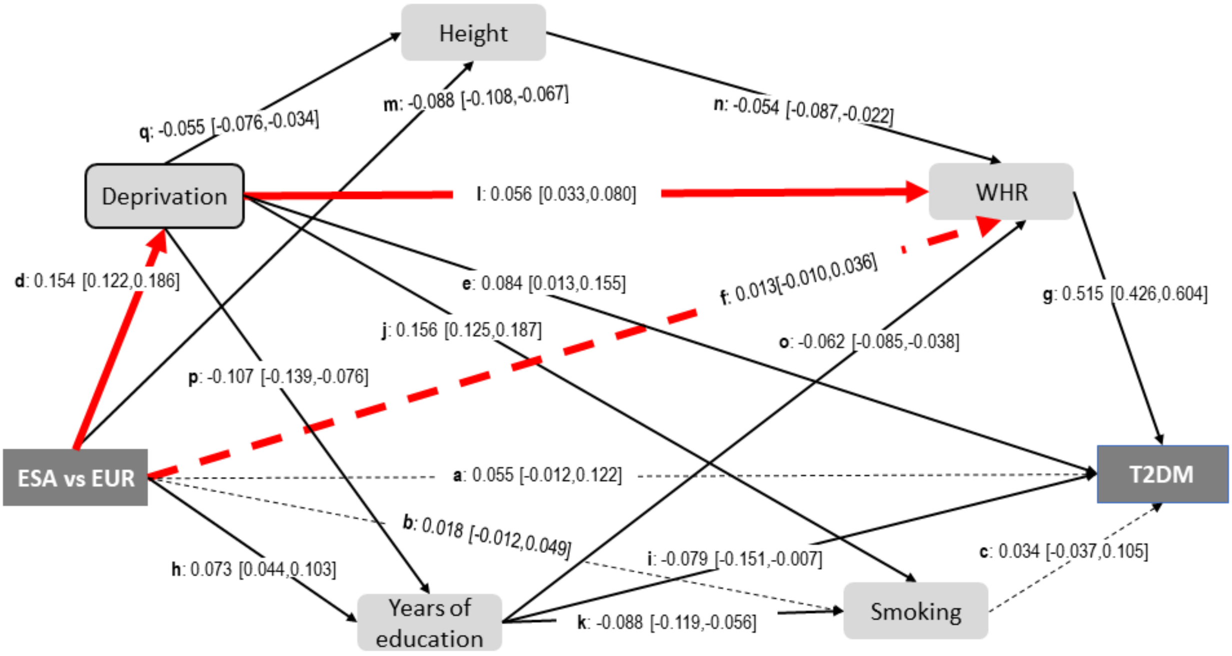

ESM Figure 12: Diagram of mediational model between Mixed Europeans/South Asian versus South Asians in type 2 diabetes prevalence, using five mediators (smoking, deprivation, WHR, height, and years of education) and their interrelationships, with focus on the pathway ethnicity-deprivation-WHR. The dashed arrows indicate non-significant association and the numbers are standardised estimates, age and sex adjusted. When breaking down the component parts (red arrows), we observed that ethnicity was strongly associated with deprivation (β_{std}=0.154, 95% CI 0.122 to 0.186), which in turn had a marked impact on WHR (β_{std}=0.056, 95% CI 0.033 to 0.080), attenuating any effect of ethnicity itself (β_{std}=0.013, 95% CI -0.010 to 0.036). Thus, a large part (37%) of the effect of ethnicity on the WHR in ESA versus Europeans was mediated via deprivation.

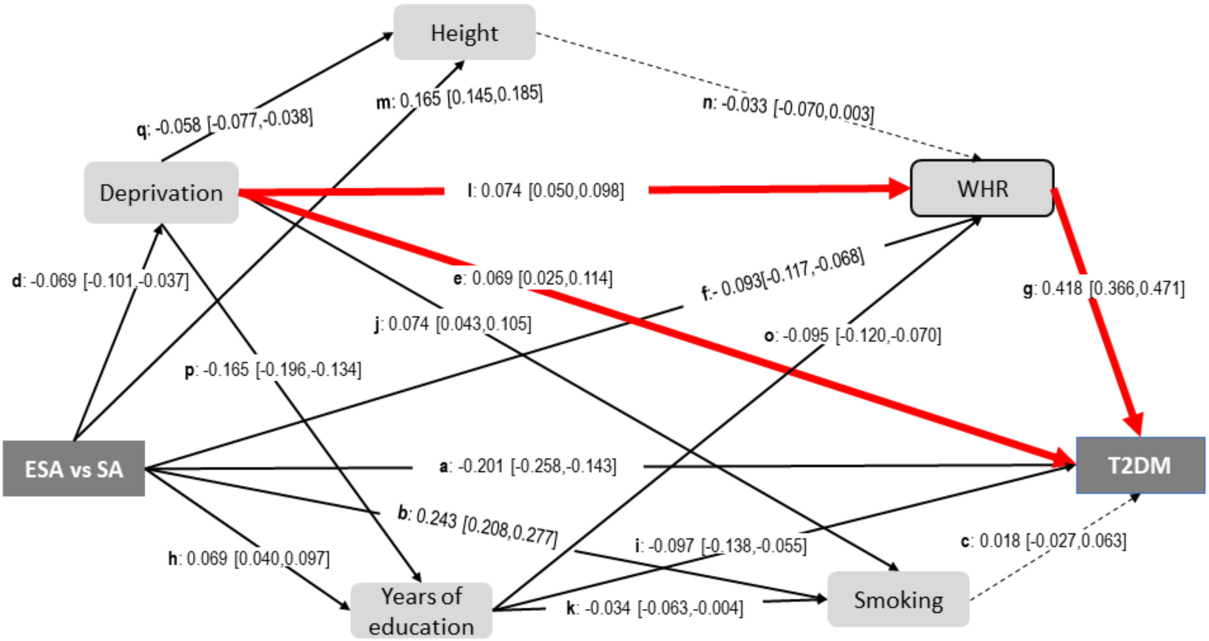

ESM Figure 13: Diagram of mediational model between Mixed Europeans/South Asian versus South Asians in type 2 diabetes prevalence, using five mediators (smoking, deprivation, WHR, height, and years of education) and their interrelationships, with focus on the pathway deprivation-WHR-T2DM. The dashed arrows indicate non-significant association and the numbers are standardised estimates, age and sex adjusted. When breaking down the component parts (red arrows), we observed that there was a modest association of ethnicity with both deprivation (β_{std}=-0.069, 95% CI -0.101 to -0.037) and WHR (β_{std}=-0.093, 95% CI -0.117 to -0.068), with the latter having a greater association on diabetes (β_{std}=0.418, 95% CI 0.366 to 0.471) than deprivation itself (β_{std}=0.069, 95% CI 0.025 to 0.114). Thus, part (24%) of the effect of deprivation in accounting for the excess prevalence of T2DM in ESA versus SA was mediated via WHR.

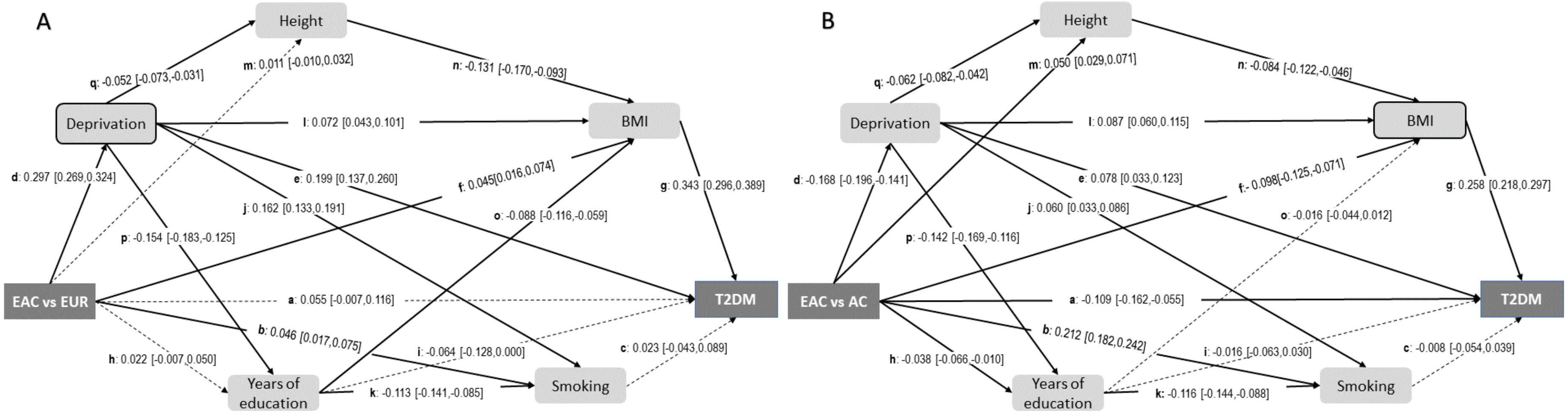

			% Mediated by													
Ethnicity	**Total effect**	**Direct effect**	**Smoking**	**Deprivation**	**BMI**	**Education**	Deprivation Smoking	Education Smoking	Deprivation BMI	Height BMI	Education BMI	Deprivation Education	Deprivation Education Smoking	Deprivation Height BMI	Deprivation Education BMI	**Total**
Pathway		(a)	(b-c)	(d-e)	(f-g)	(h-i)	(d-h-c)	(i-j-c)	(d-l-g)	(m-n-g)	(h-o-g)	(d-p-i)	(d-p-k-c)	(d-q-n-g)	(d-p-o-g)	
EAC vs EUR	0.141	0.055	1	42	11	-1	1	0	5	0	-1	2	0	1	1	**61***

			% Mediated by													
Ethnicity	**Total effect**	**Direct effect**	**Smoking**	**Deprivation**	**BMI**	**Education**	Deprivation Smoking	Education Smoking	Deprivation BMI	Height BMI	Education BMI	Deprivation Education	Deprivation Education Smoking	Deprivation Height BMI	Deprivation Education BMI	**Total**
Pathway		(a)	(b-c)	(d-e)	(f-g)	(h-i)	(d-h-c)	(i-j-c)	(d-l-g)	(m-n-g)	(h-o-g)	(d-p-i)	(d-p-k-c)	(d-q-n-g)	(d-p-o-g)	
EAC vs AC	-0.154	-0.109	1	9	16	0	0	0	2	1	0	0	0	0	0	**29**

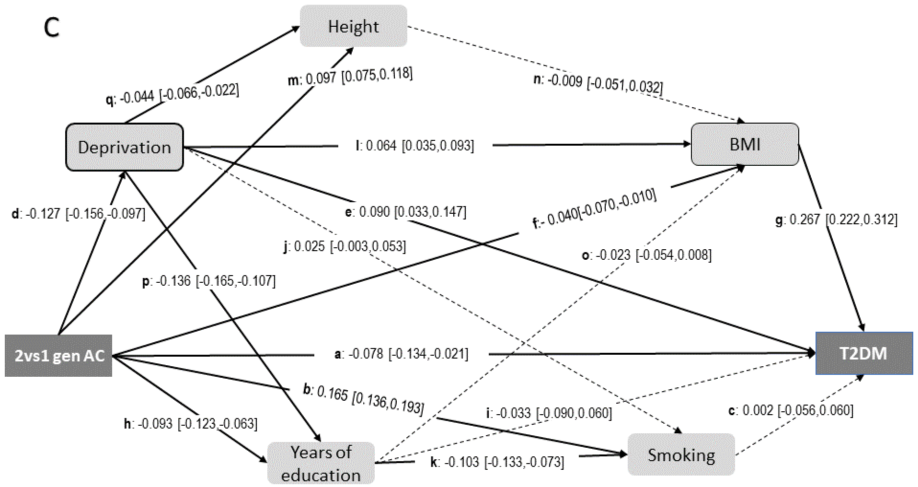

			% Mediated by													
Generation	Total effect	Direct effect	Smoking	Deprivation	BMI	Education	Deprivation Smoking	Education Smoking	Deprivation BMI	Height BMI	Education BMI	Deprivation Education	Deprivation Education Smoking	Deprivation Height BMI	Deprivation Education BMI	Total
Pathway		(a)	(b-c)	(d-e)	(f-g)	(h-i)	(d-h-c)	(i-j-c)	(d-l-g)	(m-n-g)	(h-o-g)	(d-p-i)	(d-p-k-c)	(d-q-n-g)	(d-p-o-g)	
2vs1 AC	-0.1	-0.078	0	11	11	-3	0	0	2	1	0	0	0	0	0	**22**

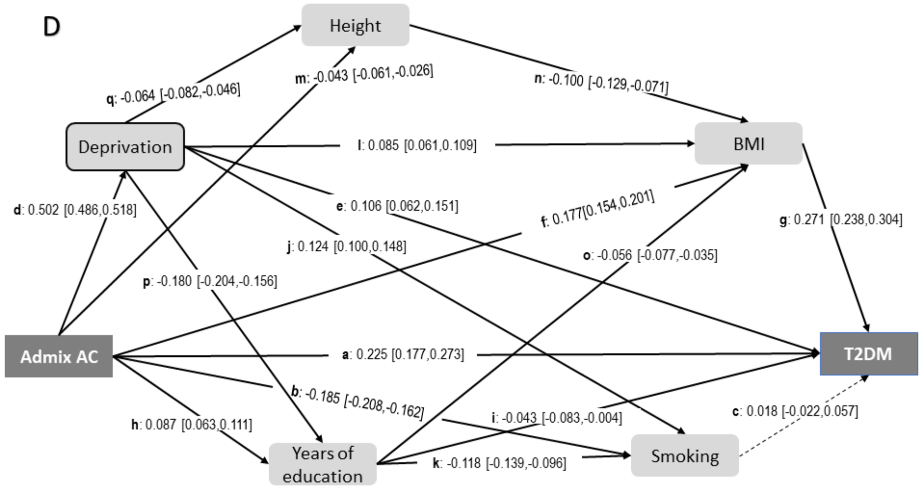

			% Mediated by													
Admixture	Total effect	Direct effect	Smoking	Deprivation	BMI	Education	Deprivation Smoking	Education Smoking	Deprivation BMI	Height BMI	Education BMI	Deprivation Education	Deprivation Education Smoking	Deprivation Height BMI	Deprivation Education BMI	Total
Pathway		(a)	(b-c)	(d-e)	(f-g)	(h-i)	(d-h-c)	(i-j-c)	(d-l-g)	(m-n-g)	(h-o-g)	(d-p-i)	(d-p-k-c)	(d-q-n-g)	(d-p-o-g)	
AC	0.346	0.225	0	18	12	0	0	0	6	0	0	0	0	0	0	**35***

ESM Figure 14: Diagrams of mediational model between African Caribbean ethnic groups (A, B), generations (C) and level of admixture (D) and the proportion of the observed difference in type 2 diabetes prevalence, which is explained by five mediators (smoking, deprivation, BMI, height and years of education) and their interrelationships. The dashed arrows indicate non-significant association and the numbers are standardised estimates, age and sex adjusted. The mediated percentages shown are rounded to the nearest integer and for this reason they might not be added up to the total (*).

A

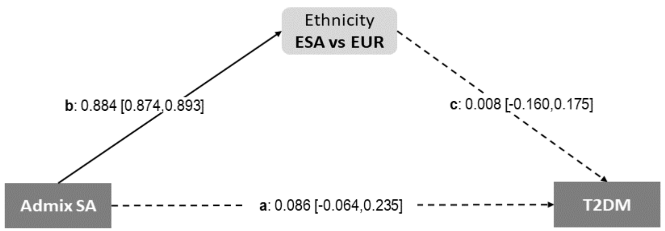

Admixture	Total effect	Direct effect	% Mediated by Ethnicity ESA vs EUR
Pathway		(a)	(b-c)
SA	0.099	0.086	**13**

B

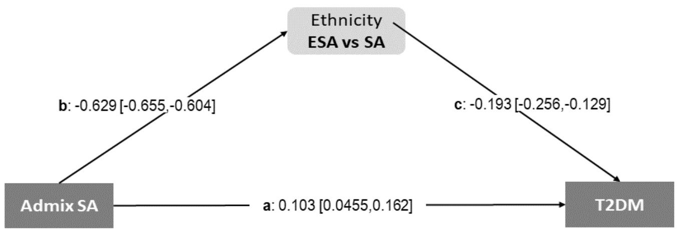

Admixture	Total effect	Direct effect	% Mediated by Ethnicity ESA vs SA
Pathway		(a)	(b-c)
SA	0.224	0.103	**54**

C

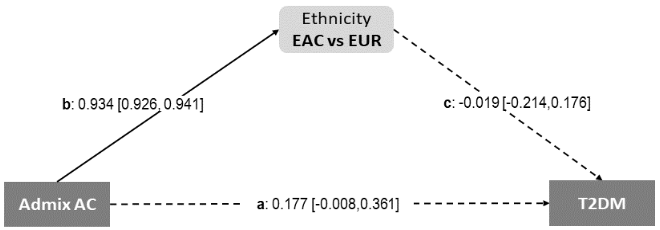

Admixture	Total effect	Direct effect	% Mediated by Ethnicity EAC vs EUR
Pathway		(a)	(b-c)
AC	0.167	0.177	**-12**

D

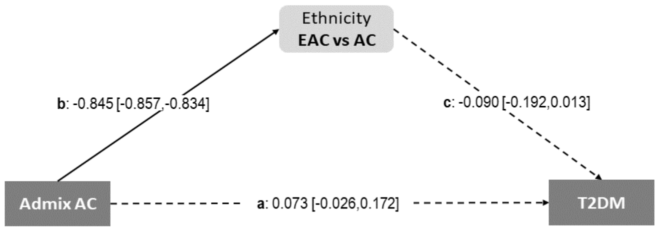

Admixture	Total effect	Direct effect	% Mediated by Ethnicity EAC vs AC
Pathway		(a)	(b-c)
AC	0.159	0.073	**54**

ESM Figure 15: Diagrams of mediational model between South Asian (A, B) and African Caribbean (C, D) level of admixture and the proportion of the observed difference in type 2 diabetes prevalence, which is explained by self-reported ethnicity in mixed vs Europeans (A, C) and in mixed vs South Asians (B) /African Caribbeans (D). The dashed arrows indicate non-significant association and the numbers are standardised estimates, age and sex adjusted.

Distribution of admixture

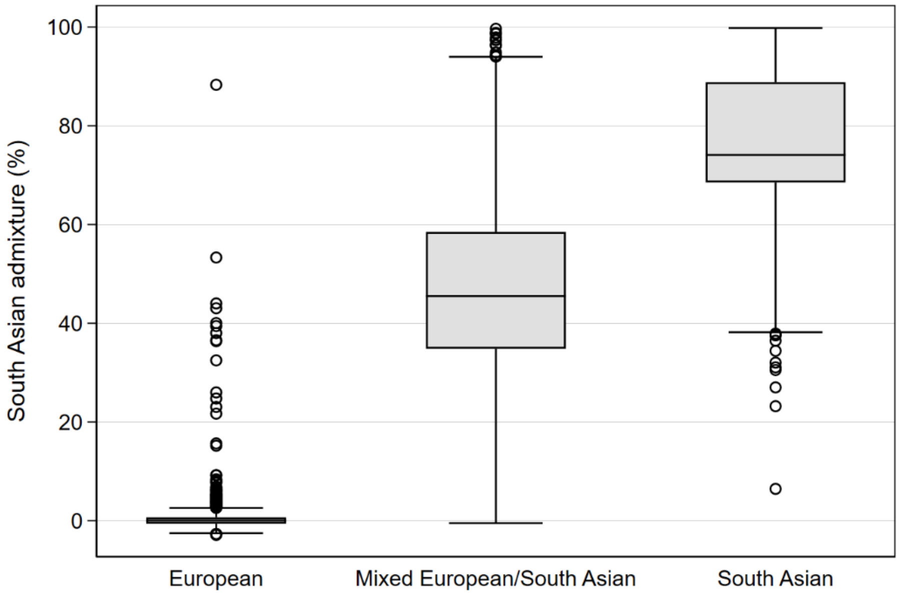

ESM Figure 16: Boxplot of South Asian admixture (%) by self-reported ethnicity

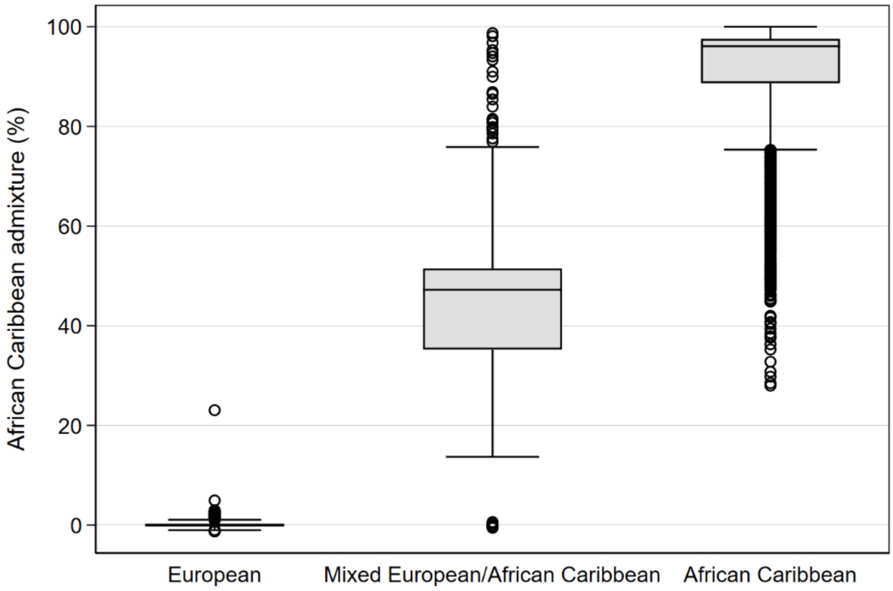

ESM Figure 17: Boxplot of African Caribbean admixture (%) by self-reported ethnicity

Regression models and sensitivity analysis

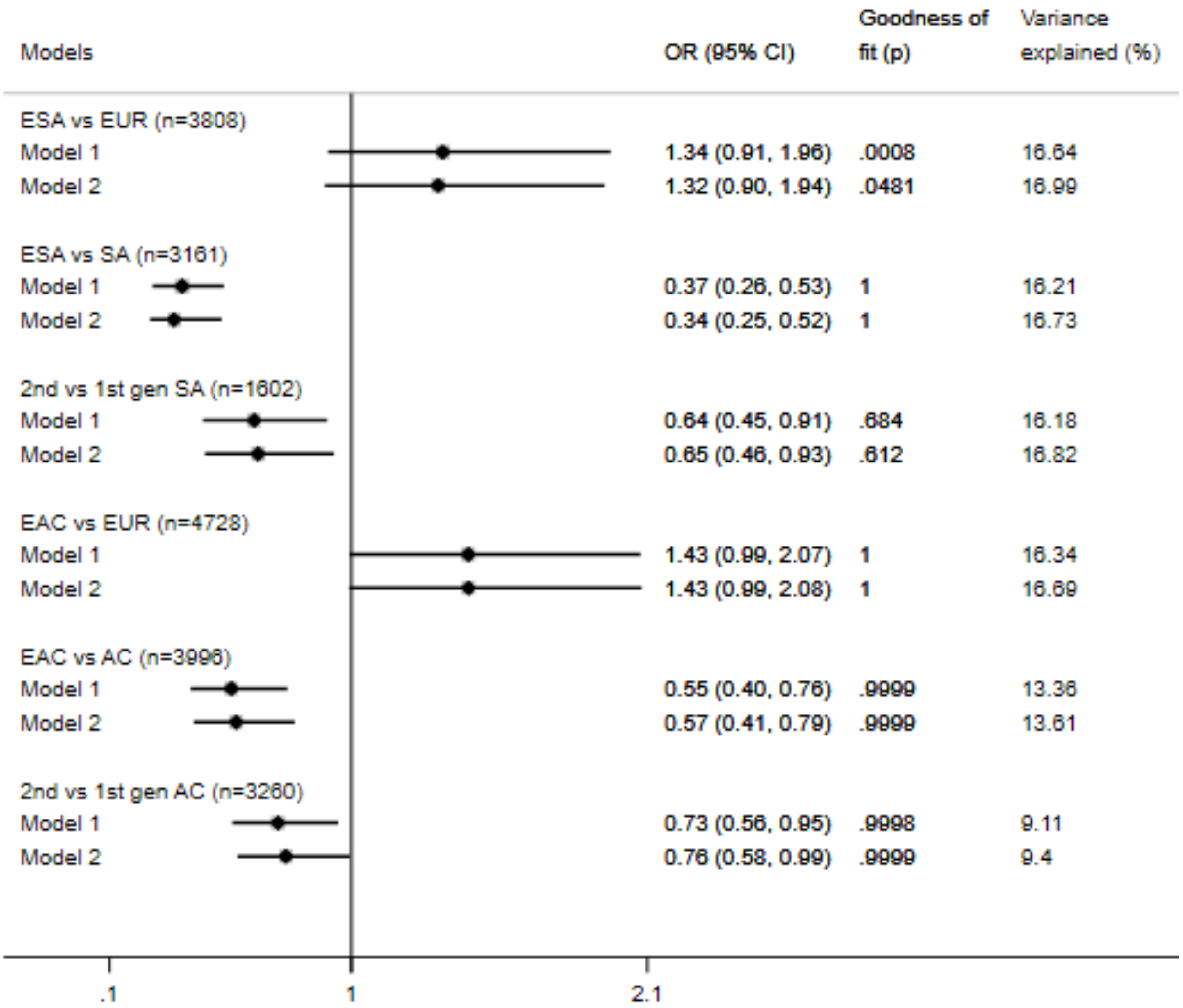

Model 1: Ethnicity/generations+ age +sex +WHR (for SA)/BMI (for AC) +deprivation +smoking+height+years of education
Model 2: Model 1 +physical activity + scores for healthy diet pattern+scores for unhealthy diet pattern

ESM Figure 18: Forest plots of multivariate regression models for diabetes adding diet and physical activity - sensitivity analysis. Sensitivity analysis was conducted on full case data for diet and physical activity.

Models	OR (95% CI)	Goodness of fit (p)	Variance explained (%)
ESA vs EUR (n=2451)			
Model 1	1.33 (0.81, 2.18)	0.354	6.9
Model 2	1.25 (0.74, 2.09)	<0.001	16.7
Model 3	1.19 (0.72, 1.97)	0.128	7.9
Model 4	1.33 (0.81, 2.18)	0.087	7.3
Model 5	1.27 (0.77, 2.10)	0.017	7.2
Model 6	1.41 (0.86, 2.32)	0.172	7.6
Model 7	1.27 (0.77, 2.08)	0.001	7.9
ESA vs SA (n=1239)			
Model 1	0.33 (0.20, 0.54)	0.314	8.6
Model 2	0.37 (0.22, 0.61)	0.997	15.7
Model 3	0.33 (0.20, 0.54)	0.860	9.8
Model 4	0.33 (0.20, 0.54)	0.289	8.6
Model 5	0.35 (0.21, 0.58)	0.595	8.8
Model 6	0.33 (0.20, 0.54)	0.452	10.6
Model 7	0.34 (0.21, 0.56)	0.226	9.5
2nd vs 1st gen SA (n=664)			
Model 1	0.84 (0.46, 1.53)	0.609	7.5
Model 2	0.89 (0.48, 1.66)	0.977	12.7
Model 3	0.85 (0.46, 1.56)	0.960	8.5
Model 4	0.82 (0.45, 1.50)	0.735	8.1
Model 5	0.84 (0.46, 1.55)	0.250	7.7
Model 6	0.78 (0.42, 1.44)	0.390	10.8
Model 7	0.87 (0.47, 1.60)	0.100	8.3

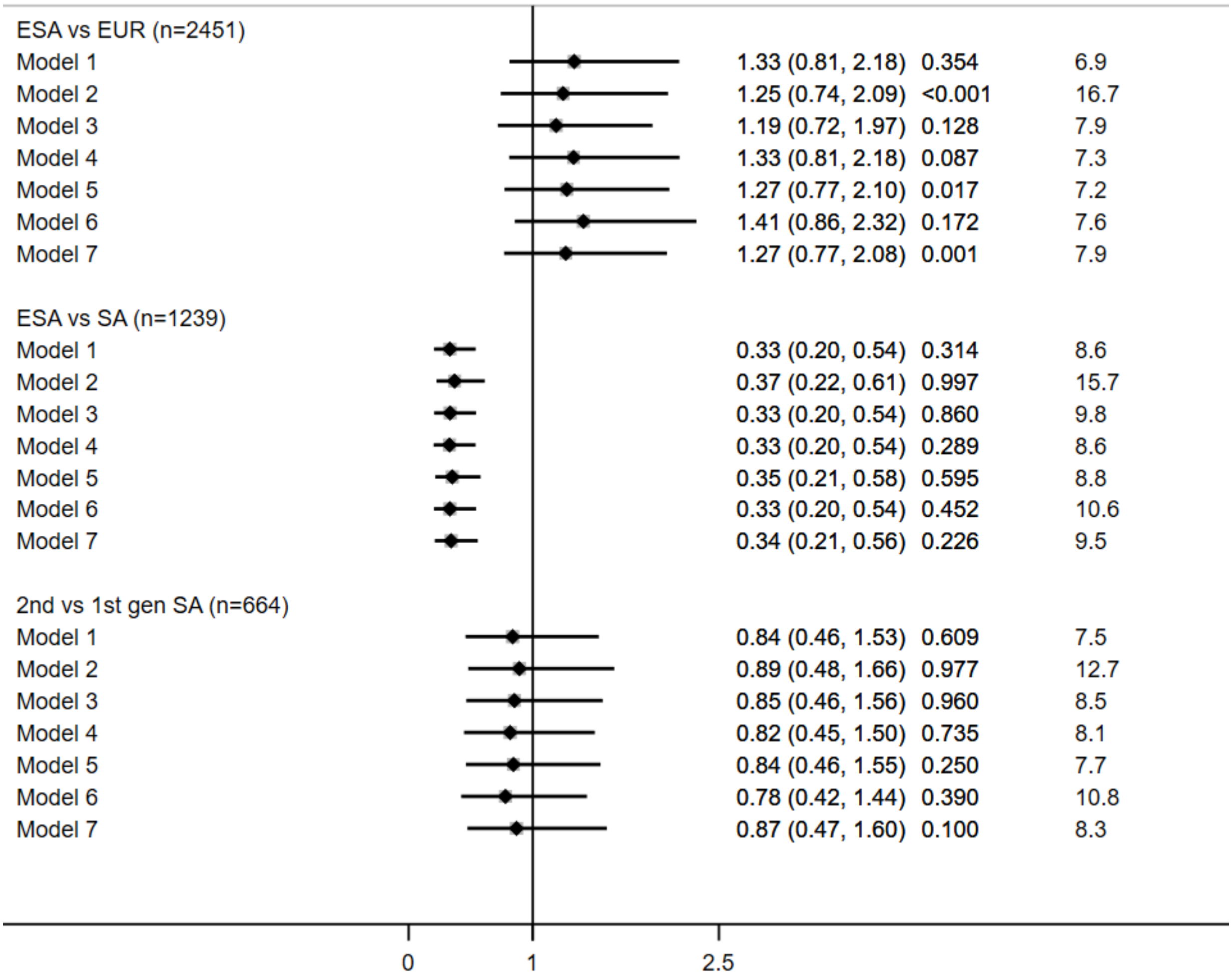

Model 1: Ethnicity/generations+ age +sex
Model 2: Model 1 +WHR
Model 3: Model 1 +deprivation
Model 4: Model 1 +smoking
Model 5: Model 1 +height
Model 6: Model 1 +years of education
Model 7: Model 1 +birth weight

ESM Figure 19: Forest plots of multivariate regression models for diabetes in South Asians adding birth weight - sensitivity analysis. Sensitivity analysis was conducted on full case data for birth weight.

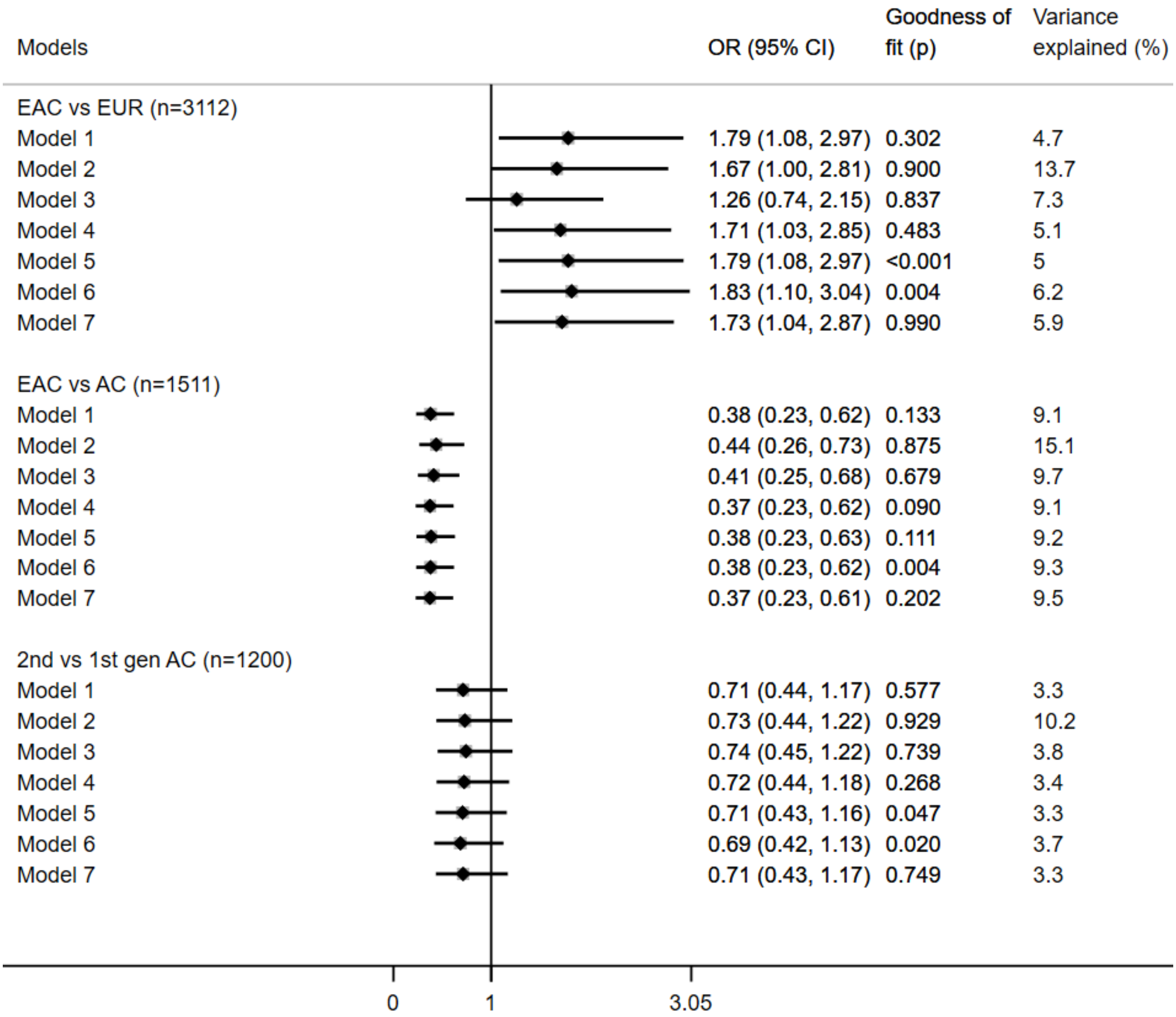

Model 1: Ethnicity/generations+ age +sex
Model 2: Model 1 +WHR
Model 3: Model 1 +deprivation
Model 4: Model 1 +smoking
Model 5: Model 1 +height
Model 6: Model 1 +years of education
Model 7: Model 1 +birth weigh

ESM Figure 20: Forest plots of multivariate regression models for diabetes in African Caribbeans adding birth weight- sensitivity analysis. Sensitivity analysis was conducted on full case data for birth weight.

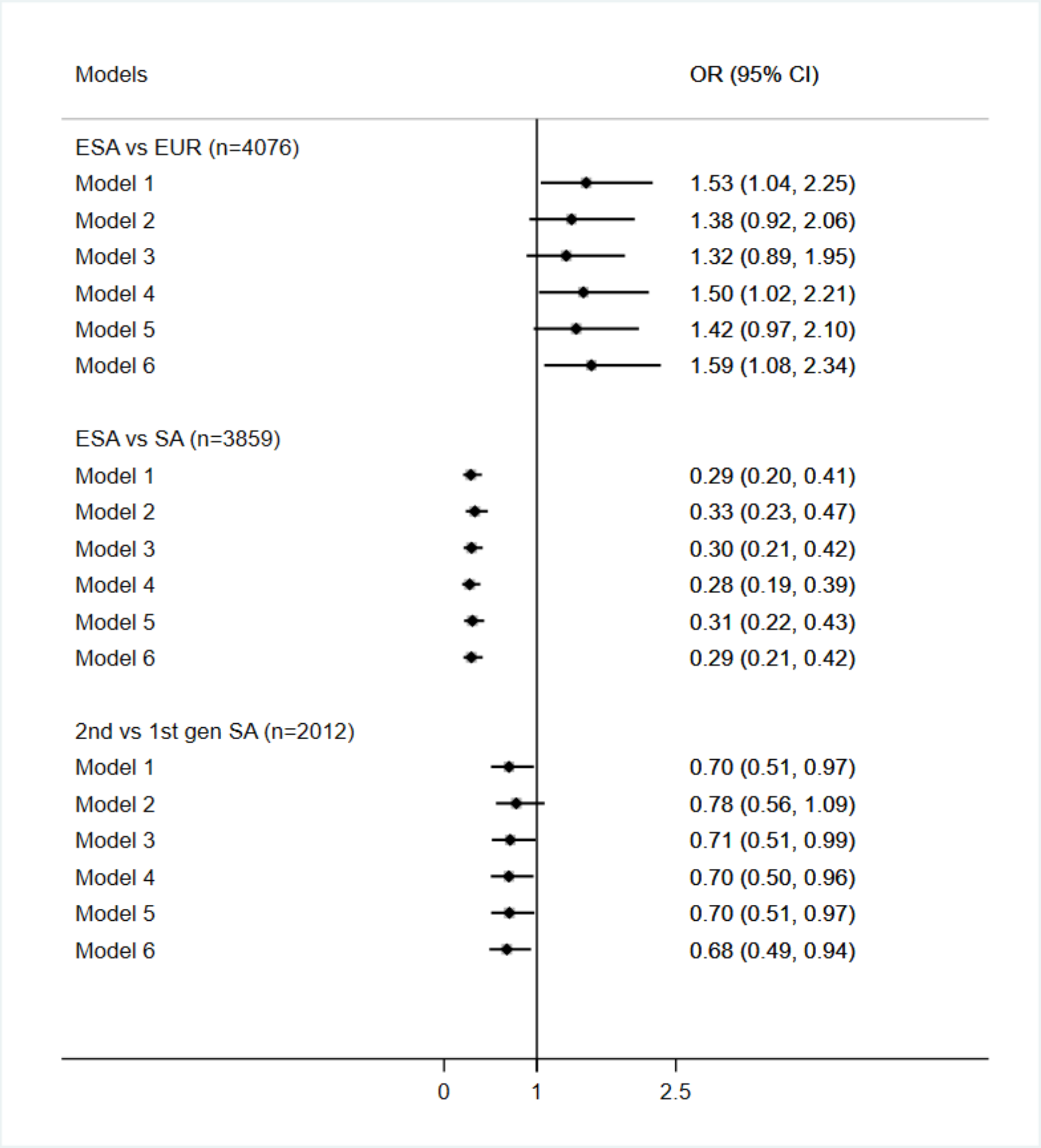

Model 1: Ethnicity/generations+ age +sex
Model 2: Model 1 +WHR
Model 3: Model 1 +deprivation
Model 4: Model 1 +smoking
Model 5: Model 1 +height
Model 6: Model 1 +years of education

ESM Figure 21: Forest plots of multivariate regression models for "Known" type 2 diabetes in South Asians. Multivariate logistic regression was used to examine the contribution of each different risk factor /determinant in the association of ethnicity with type 2 diabetes.

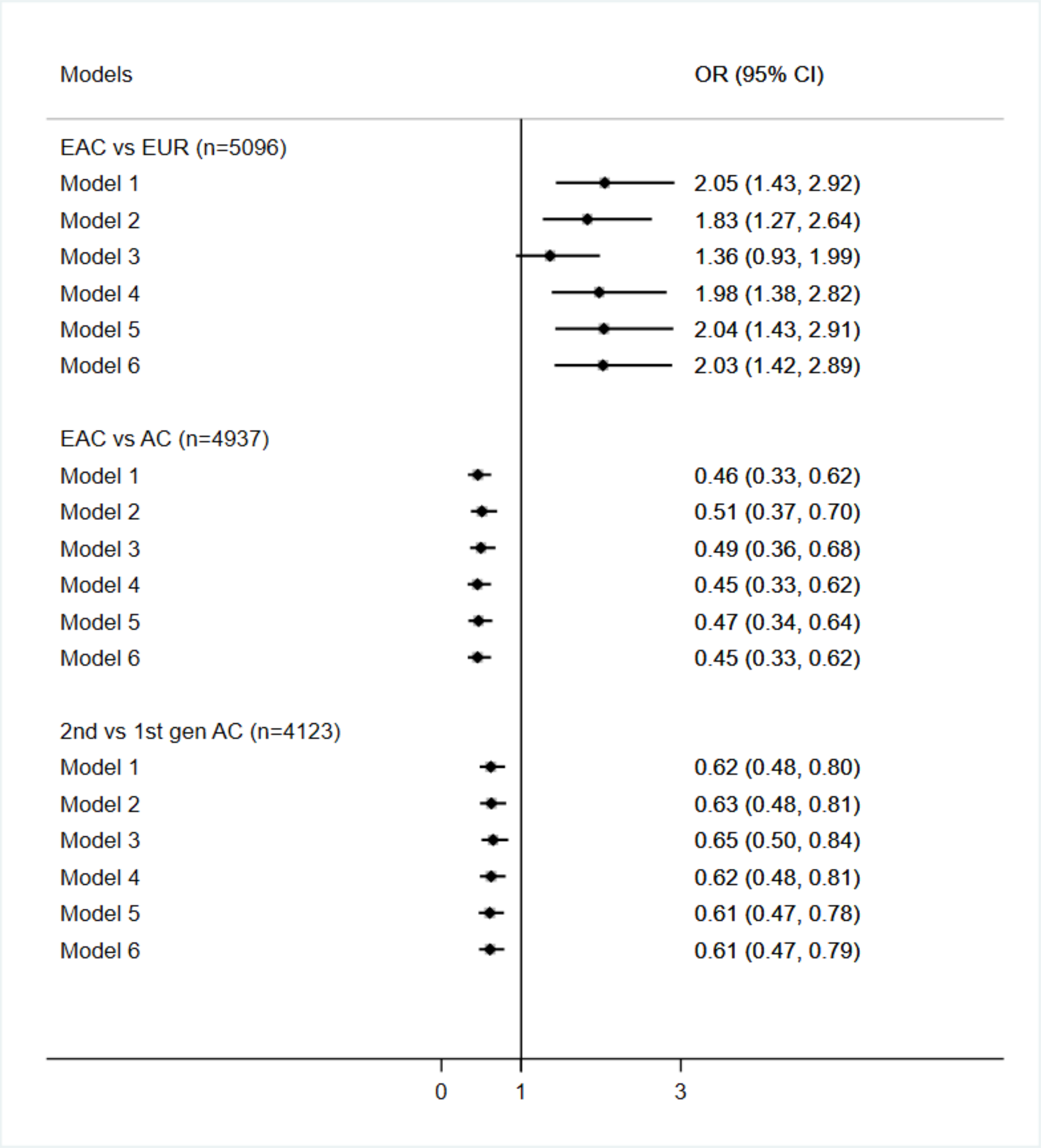

Model 1: Ethnicity/generations+ age +sex
Model 2: Model 1 +BMI
Model 3: Model 1 +deprivation
Model 4: Model 1 +smoking
Model 5: Model 1 +height
Model 6: Model 1 +years of education

ESM Figure 22: Forest plots of multivariate regression models for "Known" type 2 diabetes in African Caribbeans. Multivariate logistic regression was used to examine the contribution of each different risk factor /determinant in the association of ethnicity with type 2 diabetes.

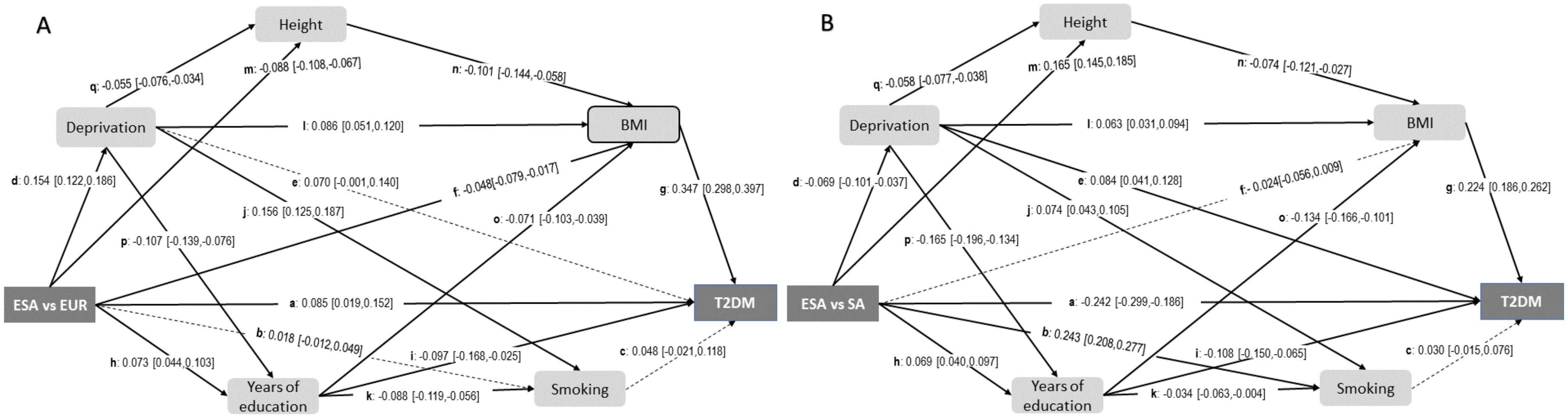

			% Mediated by													
Ethnicity	**Total effect**	**Direct effect**	**Smoking**	**Deprivation**	**BMI**	**Education**	Deprivation Smoking	Education Smoking	Deprivation BMI	Height BMI	Education BMI	Deprivation Education	Deprivation Education Smoking	Deprivation Height BMI	Deprivation Education BMI	**Total**
Pathway		(a)	(b-c)	(d-e)	(f-g)	(h-i)	(d-h-c)	(i-j-c)	(d-l-g)	(m-n-g)	(h-o-g)	(d-p-i)	(d-p-k-c)	(d-q-n-g)	(d-p-o-g)	
ESA vs EUR	0.082	0.085	1	13	-21	-9	1	0	6	4	-2	2	0	0	0	**-4***

			% Mediated by													
Ethnicity	**Total effect**	**Direct effect**	**Smoking**	**Deprivation**	**BMI**	**Education**	Deprivation Smoking	Education Smoking	Deprivation BMI	Height BMI	Education BMI	Deprivation Education	Deprivation Education Smoking	Deprivation Height BMI	Deprivation Education BMI	**Total**
Pathway		(a)	(b-c)	(d-e)	(f-g)	(h-i)	(d-h-c)	(i-j-c)	(d-l-g)	(m-n-g)	(h-o-g)	(d-p-i)	(d-p-k-c)	(d-q-n-g)	(d-p-o-g)	
ESA vs SA	-0.261	-0.242	-3	2	2	3	0	0	0	1	1	0	0	0	0	**7***

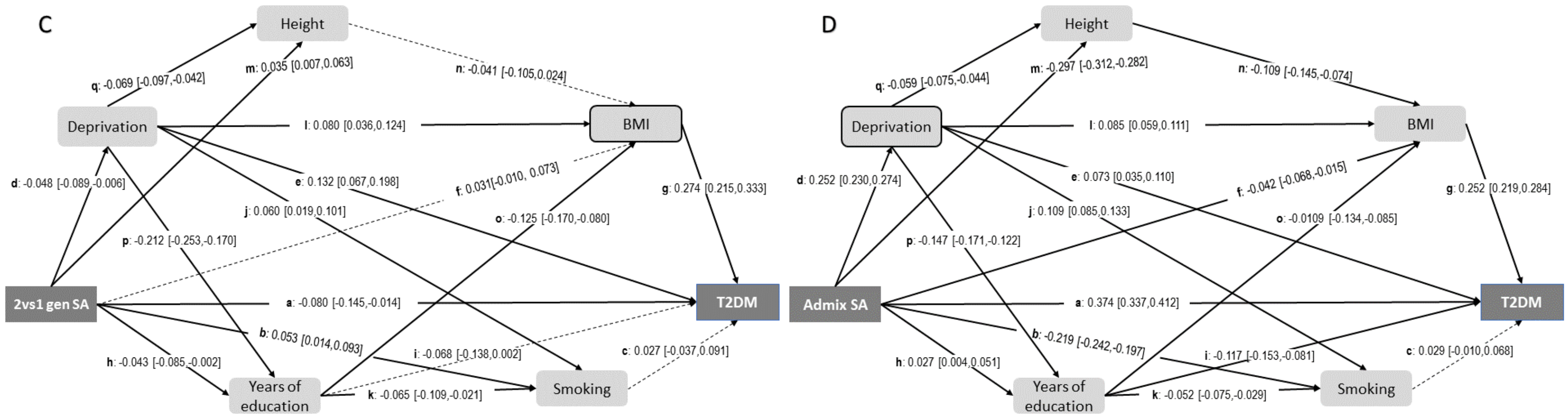

Generation	Total effect	Direct effect	% Mediated by Smoking	Deprivation	BMI	Education	Deprivation Smoking	Education Smoking	Deprivation BMI	Height BMI	Education BMI	Deprivation Education	Deprivation Education Smoking	Deprivation Height BMI	Deprivation Education BMI	Total
Pathway		(a)	(b-c)	(d-e)	(f-g)	(h-i)	(d-h-c)	(i-j-c)	(d-l-g)	(m-n-g)	(h-o-g)	(d-p-i)	(d-p-k-c)	(d-q-n-g)	(d-p-o-g)	
2vs1 SA	-0.074	-0.080	-1	8	-12	-4	0	0	1	0	-1	1	0	0	0	**-8**

Admixture	Total effect	Direct effect	% Mediated by Smoking	Deprivation	BMI	Education	Deprivation Smoking	Education Smoking	Deprivation BMI	Height BMI	Education BMI	Deprivation Education	Deprivation Education Smoking	Deprivation Height BMI	Deprivation Education BMI	Total
Pathway		(a)	(b-c)	(d-e)	(f-g)	(h-i)	(d-h-c)	(i-j-c)	(d-l-g)	(m-n-g)	(h-o-g)	(d-p-i)	(d-p-k-c)	(d-q-n-g)	(d-p-o-g)	
SA	0.392	0.374	-2	5	-3	-1	0	0	1	2	0	1	0	0	0	**5***

ESM Figure 23: Diagrams of mediational model between South Asian ethnic groups (A, B), generations (C) and level of admixture (D) and the proportion of the observed difference in type 2 diabetes prevalence, which is explained by five mediators (smoking, deprivation, BMI, height, and years of education) and their interrelationships. The dashed arrows indicate non-significant association and the numbers are standardised estimates, age and sex adjusted. The mediated percentages shown are rounded to the nearest integer and for this reason they might not be added up to the total (*).

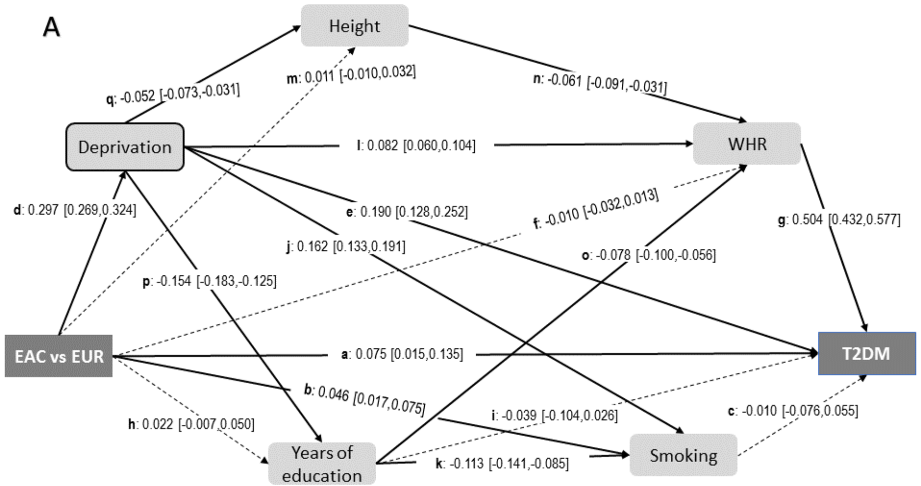

Ethnicity	Total effect	Direct effect	% Mediated by: Smoking	Deprivation	WHR	Education	Deprivation Smoking	Education Smoking	Deprivation WHR	Height WHR	Education WHR	Deprivation Education	Deprivation Education Smoking	Deprivation Height WHR	Deprivation Education WHR	Total
Pathway		(a)	(b-c)	(d-e)	(f-g)	(h-i)	(d-h-c)	(i-j-c)	(d-l-g)	(m-n-g)	(h-o-g)	(d-p-i)	(d-p-k-c)	(d-q-n-g)	(d-p-o-g)	
EAC vs EUR	0.14	0.075	0	40	-4	-1	0	0	9	0	-1	1	0	0	1	**46***

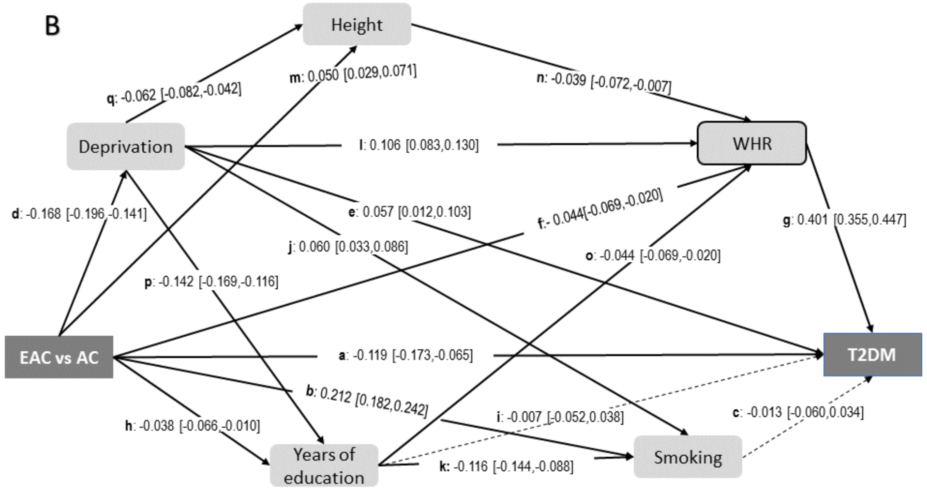

Ethnicity	Total effect	Direct effect	% Mediated by: Smoking	Deprivation	WHR	Education	Deprivation Smoking	Education Smoking	Deprivation WHR	Height WHR	Education WHR	Deprivation Education	Deprivation Education Smoking	Deprivation Height WHR	Deprivation Education WHR	Total
Pathway		(a)	(b-c)	(d-e)	(f-g)	(h-i)	(d-h-c)	(i-j-c)	(d-l-g)	(m-n-g)	(h-o-g)	(d-p-i)	(d-p-k-c)	(d-q-n-g)	(d-p-o-g)	
EAC vs AC	-0.157	-0.119	2	6	11	0	0	0	4	1	-1	0	0	0	0	**24***

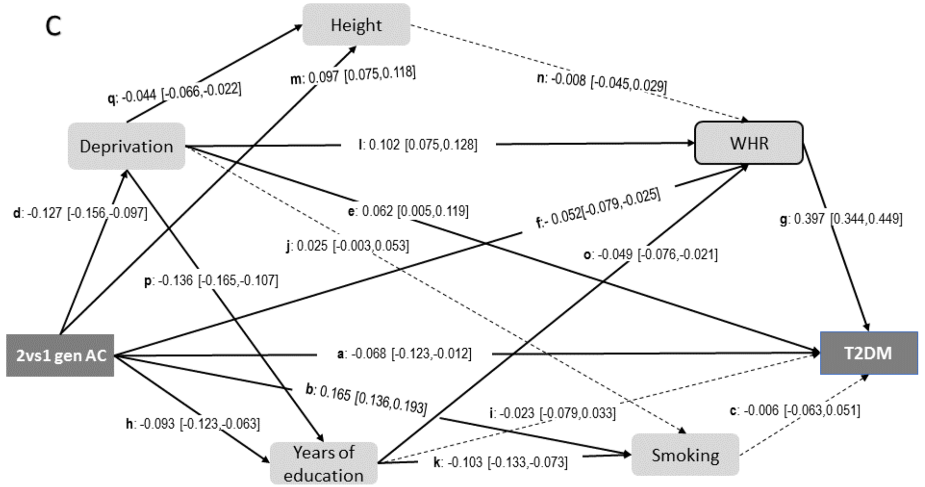

			% Mediated by													
Generation	Total effect	Direct effect	Smoking	Deprivation	WHR	Education	Deprivation Smoking	Education Smoking	Deprivation WHR	Height WHR	Education WHR	Deprivation Education	Deprivation Education Smoking	Deprivation Height WHR	Deprivation Education WHR	Total
Pathway		(a)	(b-c)	(d-e)	(f-g)	(h-i)	(d-h-c)	(i-j-c)	(d-l-g)	(m-n-g)	(h-o-g)	(d-p-i)	(d-p-k-c)	(d-q-n-g)	(d-p-o-g)	
2vs1 AC	-0.099	-0.068	1	8	21	-2	0	0	5	0	-2	0	0	0	0	**32***

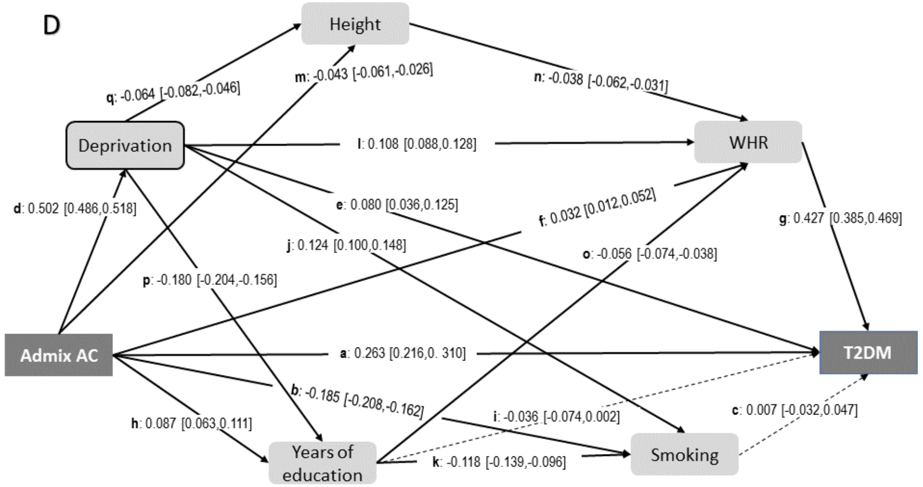

			% Mediated by													
Admixture	Total effect	Direct effect	Smoking	Deprivation	WHR	Education	Deprivation Smoking	Education Smoking	Deprivation WHR	Height WHR	Education WHR	Deprivation Education	Deprivation Education Smoking	Deprivation Height WHR	Deprivation Education WHR	Total
Pathway		(a)	(b-c)	(d-e)	(f-g)	(h-i)	(d-h-c)	(i-j-c)	(d-l-g)	(m-n-g)	(h-o-g)	(d-p-i)	(d-p-k-c)	(d-q-n-g)	(d-p-o-g)	
AC	0.341	0.263	0	12	4	-1	0	0	7	0	-1	1	0	0	1	**23**

ESM Figure 24: Diagrams of mediational model between African Caribbean ethnic groups (A, B), generations (C) and level of admixture (D) and the proportion of the observed difference in type 2 diabetes prevalence, which is explained by five mediators (smoking, deprivation, WHR, height and years of education) and their interrelationships. The dashed arrows indicate non-significant association and the numbers are standardised estimates, age and sex adjusted. The mediated percentages shown are rounded to the nearest integer and for this reason they might not be added up to the total (*).

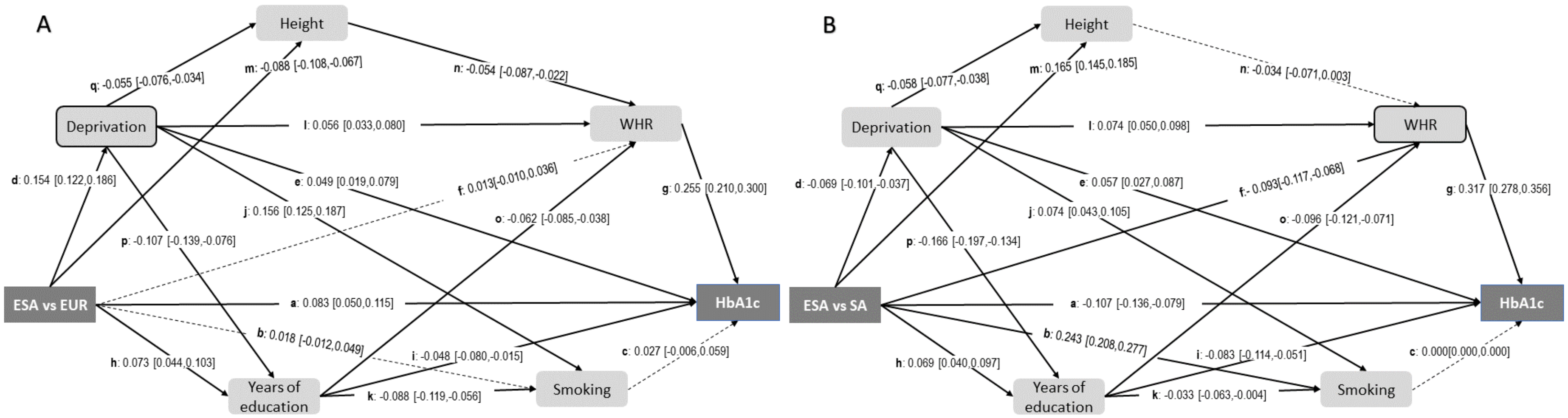

			% Mediated by													
Ethnicity	Total effect	Direct effect	Smoking	Deprivation	WHR	Education	Deprivation Smoking	Education Smoking	Deprivation WHR	Height WHR	Education WHR	Deprivation Education	Deprivation Education Smoking	Deprivation Height WHR	Deprivation Education WHR	Total
Pathway		(a)	(b-c)	(d-e)	(f-g)	(h-i)	(d-h-c)	(i-j-c)	(d-l-g)	(m-n-g)	(h-o-g)	(d-p-i)	(d-p-k-c)	(d-q-n-g)	(d-p-o-g)	
ESA vs EUR	0.095	0.083	1	8	4	-4	1	0	2	1	-1	1	0	0	0	**13**

			% Mediated by													
Ethnicity	Total effect	Direct effect	Smoking	Deprivation	WHR	Education	Deprivation Smoking	Education Smoking	Deprivation WHR	Height WHR	Education WHR	Deprivation Education	Deprivation Education Smoking	Deprivation Height WHR	Deprivation Education WHR	Total
Pathway		(a)	(b-c)	(d-e)	(f-g)	(h-i)	(d-h-c)	(i-j-c)	(d-l-g)	(m-n-g)	(h-o-g)	(d-p-i)	(d-p-k-c)	(d-q-n-g)	(d-p-o-g)	
ESA vs SA	0.153	0.107	0	3	19	4	0	0	0	1	1	1	1	0	0	**30**

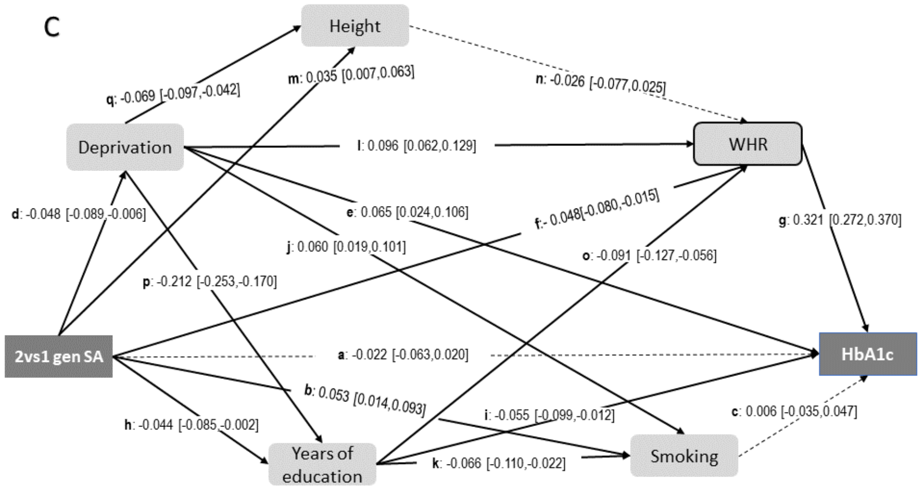

			% Mediated by													
Generation	**Total effect**	**Direct effect**	**Smoking**	**Deprivation**	**WHR**	**Education**	Deprivation Smoking	Education Smoking	Deprivation WHR	Height WHR	Education WHR	Deprivation Education	Deprivation Education Smoking	Deprivation Height WHR	Deprivation Education WHR	**Total**
Pathway		(a)	(b-c)	(d-e)	(f-g)	(h-i)	(d-h-c)	(i-j-c)	(d-l-g)	(m-n-g)	(h-o-g)	(d-p-i)	(d-p-k-c)	(d-q-n-g)	(d-p-o-g)	
2vs1 SA	-0.039	-0.022	-1	8	40	-6	0	0	4	1	-3	1	0	0	1	**44**

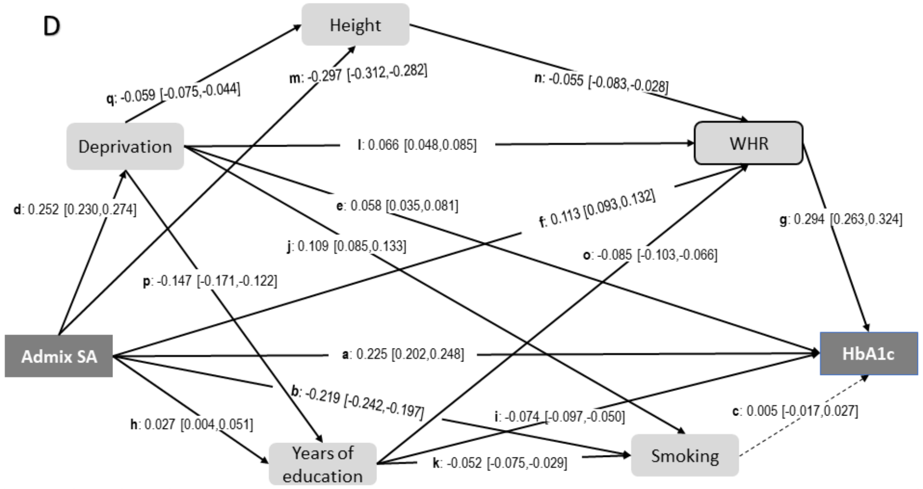

			% Mediated by													
Admixture	**Total effect**	**Direct effect**	**Smoking**	**Deprivation**	**WHR**	**Education**	Deprivation Smoking	Education Smoking	Deprivation WHR	Height WHR	Education WHR	Deprivation Education	Deprivation Education Smoking	Deprivation Height WHR	Deprivation Education WHR	**Total**
Pathway		(a)	(b-c)	(d-e)	(f-g)	(h-i)	(d-h-c)	(i-j-c)	(d-l-g)	(m-n-g)	(h-o-g)	(d-p-i)	(d-p-k-c)	(d-q-n-g)	(d-p-o-g)	
SA	0.285	0.225	0	5	11	0	0	0	2	2	0	2	0	0	0	**21***

ESM Figure 25: Diagrams of mediational model between South Asian ethnic groups (A, B), generations (C) and level of admixture (D) and the association with HbA1c levels, which is explained by five mediators (smoking, deprivation, WHR, height, and years of education) and their interrelationships. The dashed arrows indicate non-significant association and the numbers are standardised estimates, age and sex adjusted. The mediated percentages shown are rounded to the nearest integer and for this reason they might not add up to the total (*).

A

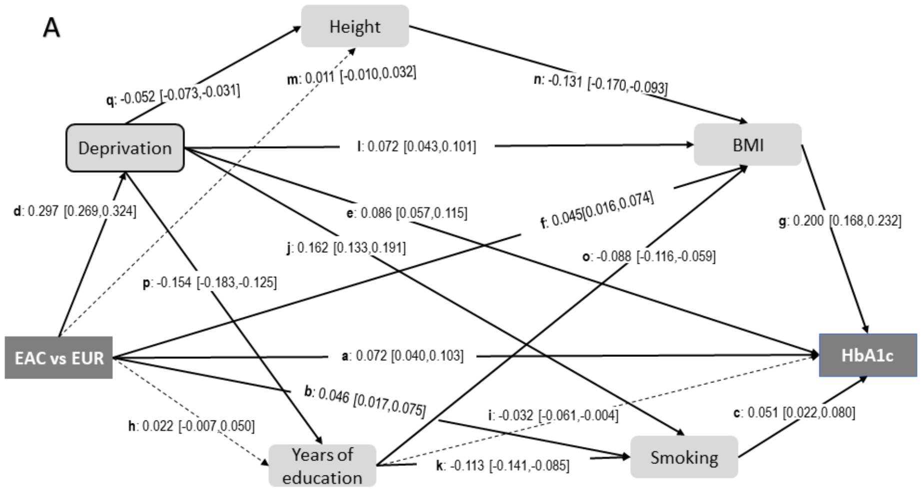

Ethnicity	Total effect	Direct effect	% Mediated by Smoking	Deprivation	BMI	Education	Deprivation Smoking	Education Smoking	Deprivation BMI	Height BMI	Education BMI	Deprivation Education	Deprivation Education Smoking	Deprivation Height BMI	Deprivation Education BMI	Total
Pathway		(a)	(b-c)	(d-e)	(f-g)	(h-i)	(d-h-c)	(i-j-c)	(d-l-g)	(m-n-g)	(h-o-g)	(d-p-i)	(d-p-k-c)	(d-q-n-g)	(d-p-o-g)	
EAC vs EUR	0.117	0.072	2	22	8	-1	2	0	4	0	0	1	0	0	1	**38***

B

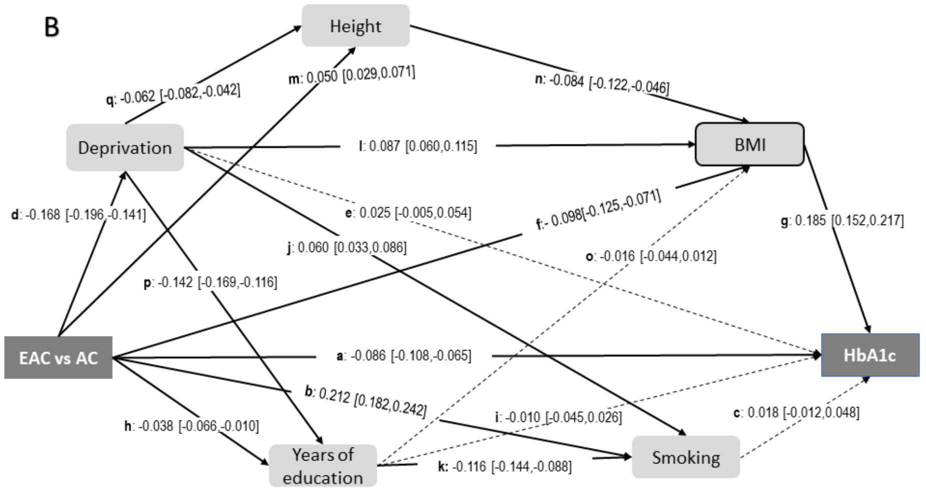

Ethnicity	Total effect	Direct effect	% Mediated by Smoking	Deprivation	BMI	Education	Deprivation Smoking	Education Smoking	Deprivation BMI	Height BMI	Education BMI	Deprivation Education	Deprivation Education Smoking	Deprivation Height BMI	Deprivation Education BMI	Total
Pathway		(a)	(b-c)	(d-e)	(f-g)	(h-i)	(d-h-c)	(i-j-c)	(d-l-g)	(m-n-g)	(h-o-g)	(d-p-i)	(d-p-k-c)	(d-q-n-g)	(d-p-o-g)	
EAC vs AC	-0.108	-0.086	-4	4	17	0	0	0	2	1	0	0	0	0	0	**20**

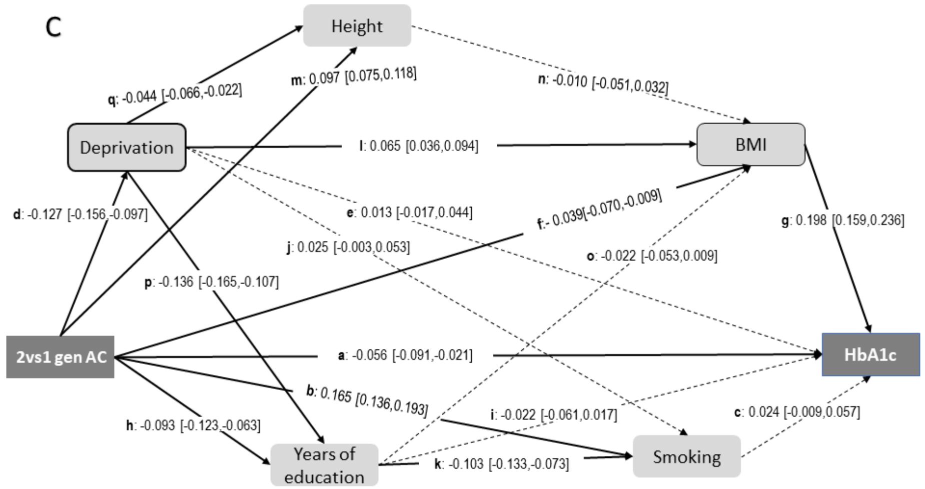

			% Mediated by													
Generation	Total effect	Direct effect	Smoking	Deprivation	BMI	Education	Deprivation Smoking	Education Smoking	Deprivation BMI	Height BMI	Education BMI	Deprivation Education	Deprivation Education Smoking	Deprivation Height BMI	Deprivation Education BMI	Total
Pathway		(a)	(b-c)	(d-e)	(f-g)	(h-i)	(d-h-c)	(i-j-c)	(d-l-g)	(m-n-g)	(h-o-g)	(d-p-i)	(d-p-k-c)	(d-q-n-g)	(d-p-o-g)	
2vs1 AC	-0.062	-0.056	-6	3	13	-3	0	0	3	0	-1	1	0	0	0	**9***

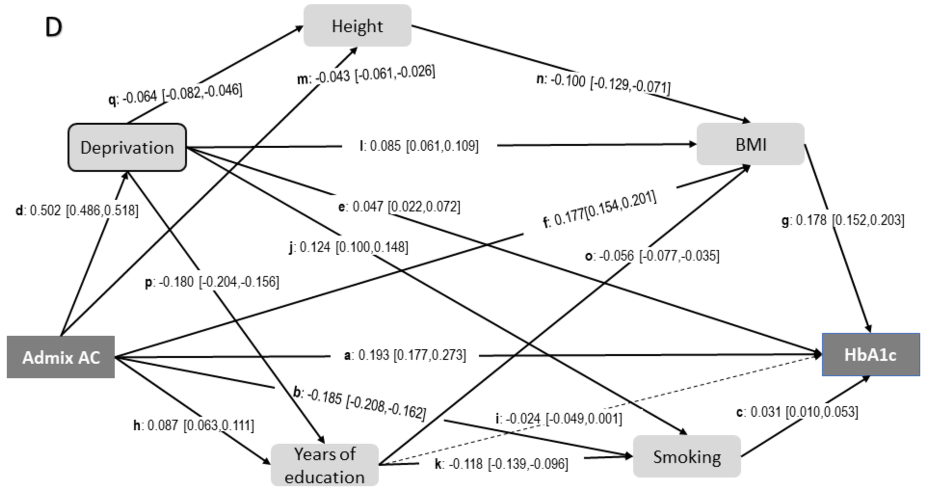

			% Mediated by													
Admixture	Total effect	Direct effect	Smoking	Deprivation	BMI	Education	Deprivation Smoking	Education Smoking	Deprivation BMI	Height BMI	Education BMI	Deprivation Education	Deprivation Education Smoking	Deprivation Height BMI	Deprivation Education BMI	Total
Pathway		(a)	(b-c)	(d-e)	(f-g)	(h-i)	(d-h-c)	(i-j-c)	(d-l-g)	(m-n-g)	(h-o-g)	(d-p-i)	(d-p-k-c)	(d-q-n-g)	(d-p-o-g)	
AC	0.251	0.193	-2	9	13	0	0	0	2	0	0	0	0	0	0	**23***

ESM Figure 26: Diagrams of mediational model between African Caribbean ethnic groups (A, B), generations (C) and level of admixture (D) and the association with HbA1c levels, which is explained by five mediators (smoking, deprivation, BMI, height and years of education) and their interrelationships. The dashed arrows indicate non-significant association and the numbers are standardised estimates, age and sex adjusted. The mediated percentages shown are rounded to the nearest integer and for this reason they might not add up to the total (*).

References

1. Bycroft C, Freeman C, Petkova D, Band G, Elliott LT, Sharp K, et al. The UK Biobank resource with deep phenotyping and genomic data. Nature. 2018;

2. Conomos MP, Miller MB, Thornton TA. Robust inference of population structure for ancestry prediction and correction of stratification in the presence of relatedness. Genet Epidemiol. 2015 May 1;39(4):276–93.

3. Heberle H, Meirelles GV, da Silva FR, Telles GP, Minghim R. InteractiVenn: a web-based tool for the analysis of sets through Venn diagrams. BMC Bioinformatics [Internet]. 2015;16(1):169. Available from: https://doi.org/10.1186/s12859-015-0611-3
